# Effect of Grape Variety and Storage Conditions on Polyphenols Stability and Antioxidant Capacities of Grape Seed Extracts

**DOI:** 10.3390/molecules31173087

**Published:** 2026-09-02

**Authors:** Pamela Georgieva, Yavor Ivanov, Zlatina Chengolova, Gjore Nakov, Tzonka Godjevargova

**Affiliations:** 1 Department Biotechnology, Burgas State University “Prof. Dr. Assen Zlatarov”, 8010 Burgas, Bulgaria; pamela.georgieva97@abv.bg (P.G.); qvor_burgas@abv.bg (Y.I.); zlatinabe4eva@abv.bg (Z.C.); 2 College of Sliven, Technical University of Sofia, 8800 Sliven, Bulgaria; gore_nakov@hotmail.com

**Keywords:** dried seed conditions, grape seed extracts, grape variety, storage conditions, polyphenols stability, antioxidant capacity, degradation kinetic, RP-HPLC

## Abstract

The utilization of grape seeds, a by-product of winemaking, and the production of valuable bioactive products from them is a very promising concept in the field of industrial waste management. The aim of this study was to investigate the effect of grape variety and storage conditions on the polyphenol stability and antioxidant capacities of grape seed extracts over a long period of time (10 months). The separated seeds from the pomace of the red grape varieties Cabernet Sauvignon and Cabernet Franc were dried at 23 °C for 10 days and at 40 °C and 60 °C for 24 h. The polyphenol extraction from all types of dried seeds was carried out under the same conditions: 70% aqueous ethanol, magnetic stirring at 600 rpm, for 3 h. Seeds dried at 40 °C yielded extracts with the highest total phenolic content. The extracts obtained from them were prepared in three physical forms: liquid concentrated form, lyophilized form and dried form at 40 °C. All the obtained extract forms were stored at 4 °C and 23 °C for 10 months. The effects of grape variety, extract physical forms and extract storage conditions on the total phenolic content, total flavonoids, procyanidins and antioxidant capacity of the extracts were studied. All parameters were determined at 0, 3, 6 and 10 months. It was found that the two types of dried extracts showed the highest stability of polyphenols at 4 °C for 10 months. The loss of total phenolic content of lyophilized Cabernet Franc GSE under these conditions was only 8.17%. All the physical forms of Cabernet Franc GSE exhibited lower losses of antioxidant activities compared to those of the Cabernet Sauvignon GSE. It was found that the reduction of antioxidant activity followed first-order kinetics. The individual compounds of the liquid and lyophilized Cabernet Franc and Cabernet Sauvignon seed extracts stored at 4 °C and 23 °C for 0 and 10 months were determined by HPLC, and their percentage loss was calculated. After 10 months of storage, the greatest degradation losses were recorded for gallic acid, procyanidin C1 and epicatechin, followed by procyanidin B3. Among the analyzed polyphenolic compounds, procyanidin B1 demonstrated the highest stability, with a loss of only 0.6%, followed by catechin and procyanidin B2, with losses of 4.02% and 2.35%, respectively. The effect of differences in the chemical composition of the two grape varieties on the stability of polyphenols and antioxidant activity of the extracts was also evaluated.

## 1. Introduction

Grape pomace is considered one of the most promising by-products for valorization. It is the primary by-product of the winemaking industry. This study contributes to reducing the negative impact on the environment, reducing food waste, implementing circular economy guidelines and enabling the development of new ingredients with potential health benefits. Globally, approximately 10–13 million tons of grape pomace are generated annually [1,2,3]. Grape pomace contains 50% skins, 25% seeds and 25% stalks [4,5]. The percentage of valuable biologically active substances is 70% in the seeds, 20% in the skins, and 10% in the stalks [6]. Grape seeds are an economically viable raw material containing biologically active polyphenols with great benefits for human health [7,8]. They mainly contain the following phenolic compounds with high antioxidant potential: (+)-catechins, (−)-epicatechin, (−)-epicatechin-3-O-gallate and dimeric and trimeric procyanidins [9,10]. The production of grape seed extract and its application as a dietary supplement and functional food additive have been a very promising direction in recent years [11,12]. They have valuable antimicrobial, antilipidemic, anti-inflammatory, antidiabetic, anticancer, and cardioprotective effects, which determine their broad potential for applications in the food, medical, pharmaceutical, and cosmetic industries [13,14].

An important issue in the preparation and storage of grape seed extracts is the achievement and preservation of high antioxidant capacity. Numerous factors influence the antioxidant potential of the obtained extract, including the grape seed variety, geographical origin and climatic growing conditions, the drying method of the grape seeds, and the storage conditions of the extracts. Several studies have confirmed that extracts obtained from seeds of different grape varieties exhibit varying polyphenolic compositions with different stabilities [15,16]. Sochorova [4] reported that the stability of phenolic compounds in the extract depends on their chemical nature and concentration. The specific polyphenolic compounds in the extract determine its stability to heat, moisture, light and oxidation [17,18]. Therefore, the polyphenolic profile of grape seeds is an important factor in the extract stability, and this factor deserves to be investigated by comparing the stability of seed extracts obtained from two different grape varieties.

Little attention is paid in the scientific literature to investigating the combined effect of grape seed variety, seed drying conditions, final physical form of the extracts and storage conditions on the stability of polyphenols in the grape seed extract for long periods. Polyphenols have unsaturated bonds, which make them unstable during long-term storage [19]. In this regard, it is important to choose appropriate conditions for drying grape seeds and for storing extracts. There are a significant number of publications investigating the drying conditions of grape pomace [19,20,21], but for grape seeds they are limited [22,23]. A number of publications show that drying grape pomace at higher temperatures above 60 °C takes less time and provides lower residual moisture, but degradation of the more unstable phenolic compounds is observed [24,25]. Gerardi et al. [21] reported that drying of pomace at 50 °C is preferable to freeze-drying because it is faster, more reproducible, and provides good storage of grape pomace. Jin et al. [26] reported that drying of grape pomace at 40 °C is preferable. The study of drying grape seeds specifically is important because their polyphenol profile is different from that of grape pomace.

It is essential to investigate the stability of polyphenols in grape seed extract during long-term storage, to determine the appropriate storage conditions and to study the degradation of individual polyphenols during storage. Many authors have published data on the storage of grape pomace extract for a maximum period of 5–6 months [20,21,25,27,28]. Only a limited number of studies have investigated the stability of grape seed extract, but the storage period is short [22,23]. The authors described that the suitable storage conditions for the dried extract are a cool place (15–25 °C) and for the liquid form, a refrigerator (4 °C) [19,23]. The degradation processes of polyphenols during longer storage periods of grape seed extracts have not been sufficiently studied. Preservation of the antioxidant activity of the extract over a long period is one of the most important goals in the production of nutritional supplements and requires further research.

In our previous publication [29], we investigated the influence of storage conditions on the stability of Syrah grape seed extract over time. In that study, the effects of the physical form of the extract, the seed-drying conditions (room temperature and 40 °C) and extract storage conditions on extract stability were monitored.

In the present study, these investigations were extended to further elucidate the factors affecting the stability of grape seed extracts. The primary objective was to investigate the effect of grape variety on the stability of grape seed extracts. For this purpose, two widely cultivated grape varieties, Cabernet Franc and Cabernet Sauvignon, were selected. Both varieties were grown in the same region and year under comparable climatic conditions. Furthermore, identical extraction and storage conditions were used for both extracts, allowing a direct comparison of their stability.

The comparison of the degradation of individual polyphenolic compounds in extracts obtained from the two grape varieties provides further insight into the influence of grape variety on extract stability during prolonged storage. In addition, the investigation of the effects of grape seed-drying conditions was expanded to provide a more comprehensive understanding of their role in determining the stability of grape seed extracts.

The aim of this study was to investigate the combined influence of grape seed variety, seed drying conditions, the physical form of the extract and storage conditions on the stability of polyphenols and the antioxidant activity of extracts from seeds of two different grape varieties over 10 months. The degradation of individual phenolic compounds in both types of extracts was studied during this period, and kinetic models were derived to describe the degradation processes.

## 2. Results

### 2.1. The Seed Moisture and GSE Yield According to Seed Drying Conditions

The grape seeds used in this study were obtained as by-products of red wine production from Cabernet Franc and Cabernet Sauvignon grape cultivars (Pomorie Wine Winery Ltd., Pomorie, Bulgaria). Both grape cultivars belong to the species *Vitis vinifera*. Cabernet Sauvignon is a naturally occurring hybrid of Cabernet Franc and Sauvignon Blanc. Although these cultivars belong to the same family, they differ in their polyphenolic composition and properties, even when cultivated under identical climatic conditions and in the same geographical location. These grape varieties were selected precisely because they are some of the most common varieties in the world for wine production [30]. Grape seeds are typically separated from the pomace in large quantities during a relatively short seasonal period. Therefore, stabilization of the separated seeds by drying is necessary, as untreated seeds are susceptible to spoilage. Drying conditions should be optimized to achieve a balance between processing costs and the final quality of the dried seeds.

In the present study, the separated seeds from the pomace were washed and dried to constant weight. Three different drying conditions were selected: drying at room temperature (23 °C) in a dark environment for 10 days, oven drying at 40 °C and oven drying at 60 °C for 24 h. The dried seeds were collected in tightly sealed dark glass containers and stored at room temperature in a dark place protected from light. The moisture content of Cabernet Franc and Cabernet Sauvignon dried seeds (all samples) was determined and is presented in Table 1.

It is obvious from Table 1 that the seeds dried at the highest temperature (60 °C) have the lowest percentage of residual moisture. The moisture content of the seeds dried at 40 °C is slightly lower than that of those dried at 23 °C, and the drying time was ten times shorter. An important task in obtaining the extracts is achieving a high yield of polyphenols. For this purpose, the isolation of the polyphenols from all seed samples is carried out using a 70:30 (*v*/*v*) ethanol–water solvent mixture with continuous stirring at 600 rpm for 3 h at room temperature. The ratio of seed powder to solvent was 1:10. Ethanol was used as the organic solvent, as it provides a high yield of the extract and is safe for human health. The obtained extracts were reduced to a final volume of 5 mL using a vacuum evaporator. The extract yield was measured using a gravimetric method. The conducted experiments demonstrated that the polyphenol yield obtained from both types of grape seeds dried at 60 °C for 24 h was the lowest compared to the yields achieved under the other two drying conditions (at 40 °C for 24 h and 23 °C for 10 days). Most likely, the higher temperature induced degradation of more sensitive polyphenolic compounds, which consequently affected the polyphenol yield. The highest extraction yield was observed in grape seeds dried at 40 °C for 24 h, probably due to the fact that the elevated temperature (40 °C) improves the extractability of polyphenols and no degradation of phenols was observed. This is why this temperature and time were chosen as the optimal drying conditions. It was found that, under all three drying conditions, the polyphenol yield of the Cabernet Sauvignon extract was about 2% higher than that of the Cabernet Franc extract.

The total phenol content (TPC) of the three liquid extracts obtained from seeds dried at 23, 40 and 60 °C was determined. It was found that the TPC of extracts from seeds dried at 23 °C and at 60 °C were 2 and 5% lower, respectively, than the TPC of the extract from seeds dried at 40 °C. The obtained results prove that the optimal seed drying conditions are 40 °C for 24 h, because they ensured a low moisture percentage, the highest polyphenol yield and the highest TPC value. Moreover, this drying approach is favored since the drying duration is roughly ten times shorter than that at 23 °C. For this reason, all subsequent experiments were performed using extracts obtained from seeds dried at 40 °C.

### 2.2. Changes in Polyphenolic Content According to Grape Seed Variety and Extract Storage Conditions over 10 Months

**Figure 1:**
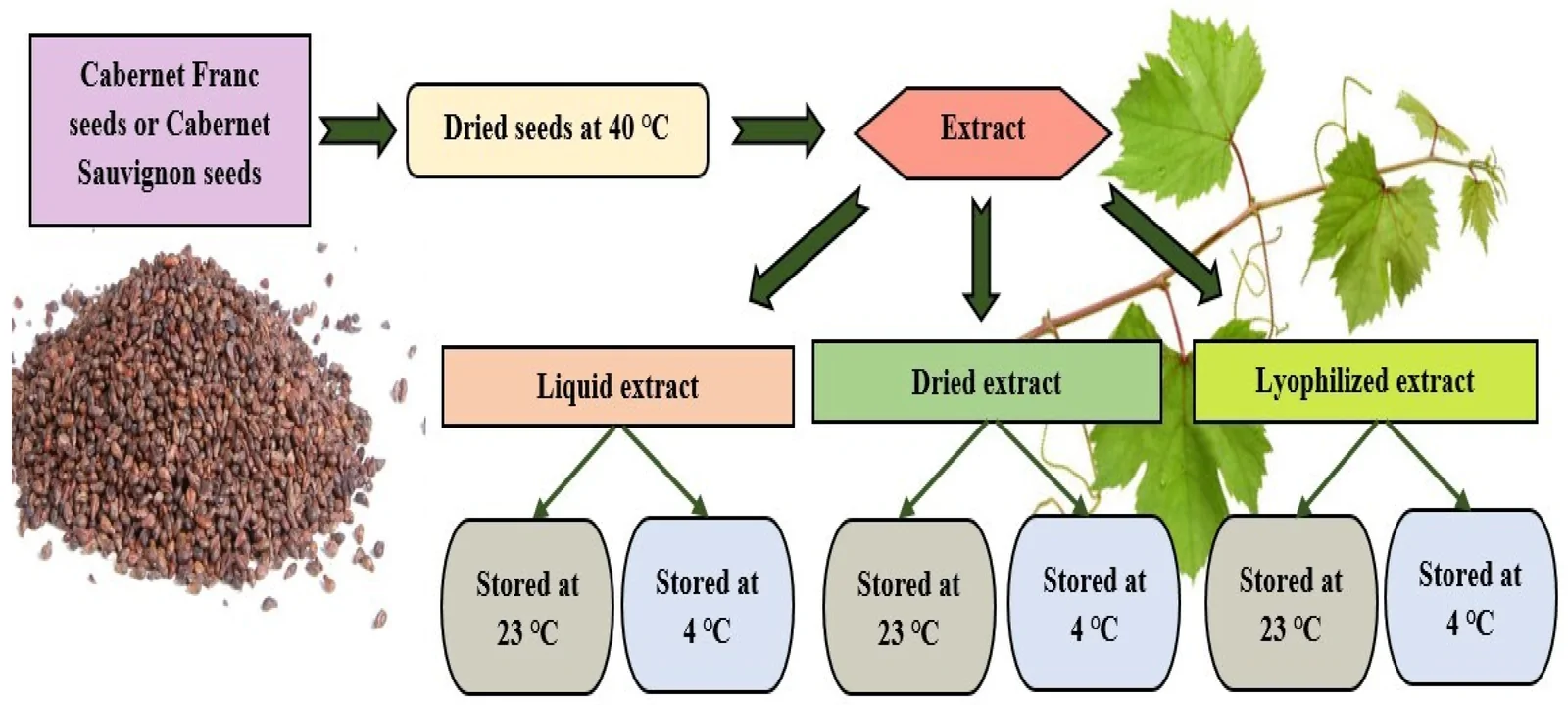
GSE forms and storage conditions.

The effects of extract form (liquid, lyophilized, and dried at 40 °C), grape seed variety, and storage conditions on the polyphenolic composition of the extracts were assessed over a 10-month period. Three extract forms were used to compare which form is more stable during storage. For this purpose, a concentrated extract of grape seeds dried at 40 °C was divided into three parts. The first portion was retained in liquid form. The second portion was dried at 40 °C for 24 h, while the third portion was lyophilized, as seen in Figure 1.

Each of the three grape seed extract (GSE) forms was stored for 10 months at 4 °C in a refrigerator and at 23 °C in a dark environment. Polyphenolic analyses were conducted after 0, 3, 6, and 10 months of storage. The values of total phenolic content (TPC), total flavonoids (TF), and procyanidins (PC) in the liquid, 40 °C-dried, and lyophilized GSEs are presented in Table 2.

The results obtained at 3, 6 and 10 months were compared with the initial values of the polyphenolic parameters. The percentage reduction (loss %) of each parameter during storage was determined and presented in brackets in Table 2.

Several relationships can be identified based on the results shown in Table 2. The grape variety from which the seeds were isolated is a very important factor determining the polyphenolic parameters of the extract. It can be seen from Table 2 that the values of TPC, TF and PC of Cabernet Franc seed extract at 0, 3, 6 and 10 months are about 5–7% higher than the results obtained for Cabernet Sauvignon seed extract. Furthermore, the losses of the studied parameters are smaller in Cabernet Franc seed extracts.

The effect of the physical form of the extracts on polyphenolic parameters was also studied. The results presented in Table 2 show that the freeze-dried extracts and the extracts dried at 40 °C exhibited higher values compared to the liquid samples. It was established that the differences between the two types of dried extracts were minimal, although the freeze-dried samples showed a slight advantage.

The data in Table 2 show that TPC exhibited the highest values, followed by TF and PC, and this trend persisted over the full duration of sample storage. The percentages shown in parentheses in Table 2 represent the reduction of each parameter during the 10-month storage period. It is noteworthy that the polyphenolic parameters of the samples stored at 4 °C exhibited considerably smaller changes compared to those stored at 23 °C, over the entire investigation period. The smallest variations in all three polyphenolic parameters were observed after 3 months of storage. At this stage, the reduction in the individual parameters ranged from 0.5 to 1.2% in the dried samples, regardless of the storage temperature (4 °C or 23 °C). A different trend was observed for the liquid samples. When stored at 4 °C, the reduction in the polyphenolic indicators in liquid samples was comparable to that observed in the dried samples. However, at 23 °C, the reduction ranged from 5 to 15%. These findings clearly demonstrate the negative effect of the aqueous medium in the liquid samples, particularly when combined with higher storage temperature. Therefore, up to 3 months of storage, the polyphenolic parameters remained relatively stable, with no significant biodegradation processes observed. Few additional changes were detected after 6 months of storage. In the dried samples (lyophilized and oven-dried extracts at 40 °C), the reduction in TPC, TF, and PC values reached up to 4% when stored at 4 °C, whereas at 23 °C, the reduction increased to approximately 12%. These results convincingly demonstrate that the dried samples stored at 4 °C retained their polyphenolic characteristics even after 6 months of storage. A similar trend was observed for the liquid concentrates stored at 4 °C, where the reduction in the measured parameters exceeded that of the dried samples by only about 4%. In contrast, the liquid samples stored at 23 °C exhibited substantially greater losses, with reductions reaching up to 25% depending on the parameter analyzed. It was also observed that TF and PC values decreased to a much greater extent than TPC values, with approximately fivefold higher reductions being recorded. The most significant differences between the stored sample values and the initial values (0 months) were observed after 10 months of storage. The reduction in the three parameters of the dried samples stored at 4 °C reached up to 20%, whereas in the liquid samples, the average reduction was approximately 30%. Once again, the liquid concentrates stored at 23 °C showed the greatest decline. The reductions in TPC values decreased by up to 25%, and TF and PC values were much more pronounced, reaching up to 70%.

### 2.3. Changes in the Individual Components of Liquid and Lyophilized Cabernet Franc and Cabernet Sauvignon GSE over 10 Months Storage

**Figure 2:**
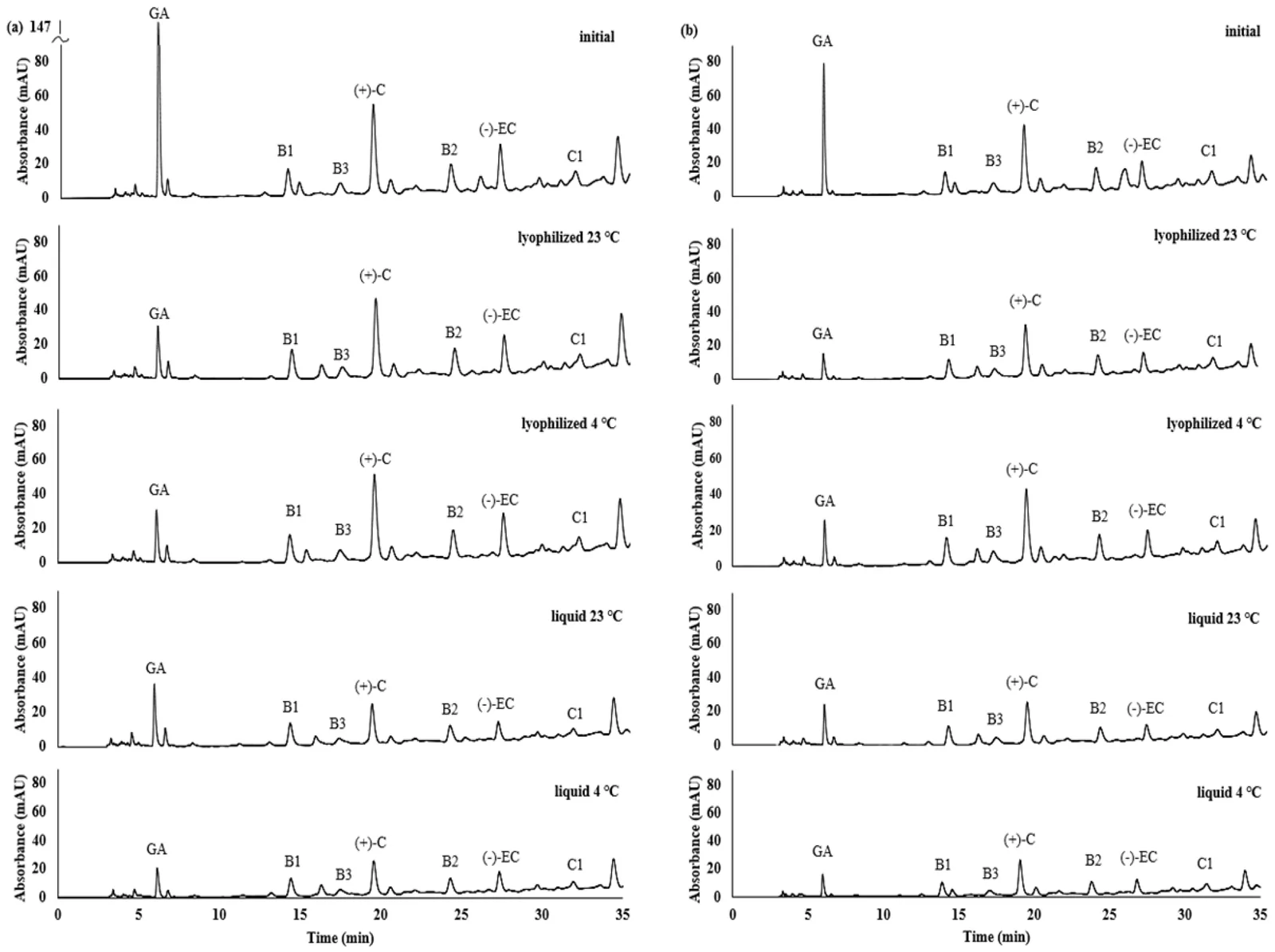
Chromatograms of Cabernet Franc (**a**) and Cabernet Sauvignon seed extract (**b**) stored for 0 and 10 months in lyophilized and liquid forms. GA—gallic acid, C—(+)-catechin, EC—(–)-epicatechin, B1—procyanidin B1, B2—procyanidin B2, B3—procyanidin B3, C1—procyanidin C1.

The study of the degradation of individual compounds in the extract during storage is an essential process to assess the degree of stability of antioxidants, especially those that have greater antioxidant potential and determine the quality of the extract. For this purpose, the individual phenolic composition of both grape seed extracts at the initial storage stage (0 months) was studied by HPLC assay. The chromatograms presented in Figure 2 demonstrate that the extracts obtained from both grape seed varieties are rich in monomeric flavan-3-ols, including (+)-catechin and (−)-epicatechin, as well as dimeric procyanidins (B1, B2, and B3), the trimeric procyanidin C1, and gallic acid.

The intensity of gallic acid absorbance in Cabernet Franc GSE is 147.8 mAU, and the same peak in Cabernet Sauvignon GSE is 80 mAU. The absorbance of GA in Cabernet Franc extract is 1.9 times higher than GA in Cabernet Sauvignon GSE. The concentrations of the identified individual compounds, expressed as mg/g dry extract weight, are presented in Table 3.

It is noteworthy that the initial concentrations of all identified individual compounds in Cabernet Franc extracts were higher than those detected in Cabernet Sauvignon extracts, with the exception of procyanidin C1. In particular, the concentrations of gallic acid, catechin, and epicatechin in Cabernet Franc extracts were approximately 1.5- or 2-fold higher compared to the corresponding values determined for Cabernet Sauvignon seed extracts.

**Figure 3:**
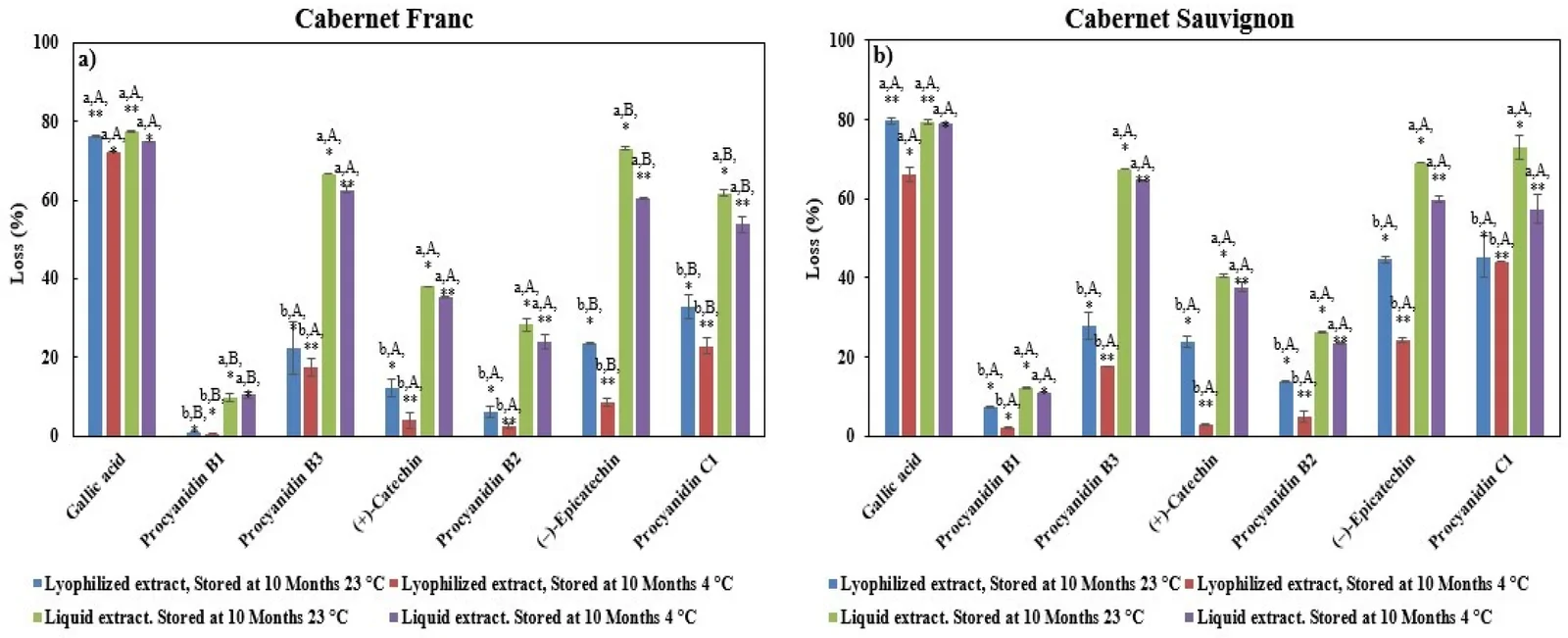
The influence of storage conditions of Cabernet Franc (**a**) and Cabernet Sauvignon (**b**) grape seed extracts on individual compound losses (%) at 10 months. Different superscripted lowercase letters indicate statistically significant differences (*p* < 0.05) among samples stored at different temperatures (4 °C and 23 °C), while values sharing the same lowercase letter are not significantly different. Different superscripted uppercase letters indicate statistically significant differences (*p* < 0.05) between the different sample forms (lyophilized and liquid), while values sharing the same uppercase letter are not significantly different. Symbols * and ** indicate significant differences between storage temperatures (4 °C and 23 °C); both correspond to *p* < 0.05, with * denoting differences for samples stored at 4 °C and ** denoting differences for samples stored at 23 °C, according to Fisher’s LSD test.

HPLC analyses were performed on lyophilized and liquid samples of Cabernet Franc and Cabernet Sauvignon GSE after storage for 10 months at 23 °C and 4 °C. Since the results obtained for the oven-dried samples at 40 °C were very similar to those of the lyophilized samples, they are not presented separately. The chromatograms obtained after storage were compared with the initial chromatographic profiles recorded at 0 months. After 10 months of storage, a substantial decrease in the intensity of the absorption peaks corresponding to the identified compounds was observed. The data presented in Table 3 demonstrate that the reduction in the concentration of the individual compounds after 10 months of storage varied considerably and was compound-specific. A large difference in the concentration of gallic acid was observed between the initial extract and the extract stored for 10 months in both types of grape seeds. In general, the reductions observed in the lyophilized samples were lower than those detected in the liquid extracts. The individual compound losses (%) at 10 months are presented in Figure 3.

For the lyophilized samples stored at 4 °C for 10 months, the identified compounds could be ranked according to their degree of reduction as follows: gallic acid (72.24%), procyanidin C1 (32.78%), (−)-epicatechin (23.54%), procyanidin B3 (22.26%), (+)-catechin (12.20%), procyanidin B2 (6.15%), and procyanidin B1 (0.91%). The percentage losses in lyophilized samples stored at room temperature are slightly higher (about 5%). The percentage losses of the identified compounds in the liquid samples were substantial, being approximately 3–4 times higher than those observed in the dried samples. The losses were also greater at 23 °C than at 4 °C. The difference in losses between the dried and liquid samples was very small only for gallic acid. In the liquid samples, the losses were substantial, reaching 77.42% for gallic acid, 61.70% for procyanidin C1, 73.17% for (−)-epicatechin, 66.67% for procyanidin B3, 37.95% for (+)-catechin, 28.30% for procyanidin B2, and 9.80% for procyanidin B1. There was also a difference in the degradation of individual compounds between the extracts obtained from the two grape varieties and stored for 10 months (Figure 3). A slight increase in the degree of degradation of individual components was found in the Cabernet Sauvignon extract compared to that in the Cabernet Franc extract.

### 2.4. Changes in Antioxidant Capacity of GSE According to Grape Seed Variety and Extract Storage Conditions over 10 Months

The antioxidant activity of all extracts stored in the dark at 23 °C and under refrigerated conditions at 4 °C was evaluated using two methods, DPPH and ABTS. The results are presented in Table 4.

The obtained results are in agreement with the polyphenolic parameters presented in Table 2. The observed differences in antioxidant (AO) activity between the liquid and dried samples followed the same trend as that established for the polyphenolic parameters. Similarly, differences between samples stored at room temperature and those stored under refrigerated conditions were observed. The obtained results at 3 months clearly demonstrate that the antioxidant activity of all investigated samples, determined by both the DPPH and ABTS assays, was better preserved during storage at 4 °C than at 23 °C. An additional noteworthy observation was that the antioxidant activity determined by the DPPH assay exhibited a lower degree of reduction during the 10-month storage period compared to the activity measured by the ABTS assay. After 6 months of storage, the antioxidant activity determined by the DPPH assay showed an average reduction of approximately 4% in all extract forms stored at 4 °C, whereas the corresponding reduction in samples stored at 23 °C reached approximately 14%. In contrast, the losses in antioxidant activity measured by the ABTS assay were more pronounced. At 4 °C, the reduction in ABTS antioxidant activity was approximately 16% for all extract forms, while storage at 23 °C resulted in reductions of approximately 20%. More significant degradation processes were observed after 10 months of storage. The percentage loss in antioxidant activity determined by the DPPH assay reached approximately 11% in samples stored at 4 °C and approximately 25% in those stored at 23 °C. The reduction in antioxidant activity measured by the ABTS assay increased considerably, reaching approximately 50% in samples stored at 4 °C and up to 70% in those stored at 23 °C. Regardless of the analytical method applied, the dried samples stored at 23 °C consistently exhibited approximately 10% lower percentage losses in antioxidant activity compared to the corresponding liquid samples.

### 2.5. Degradation Kinetics of Polyphenols on the Basis of Extract Antioxidant Capacity over 10 Months

**Figure 4:**
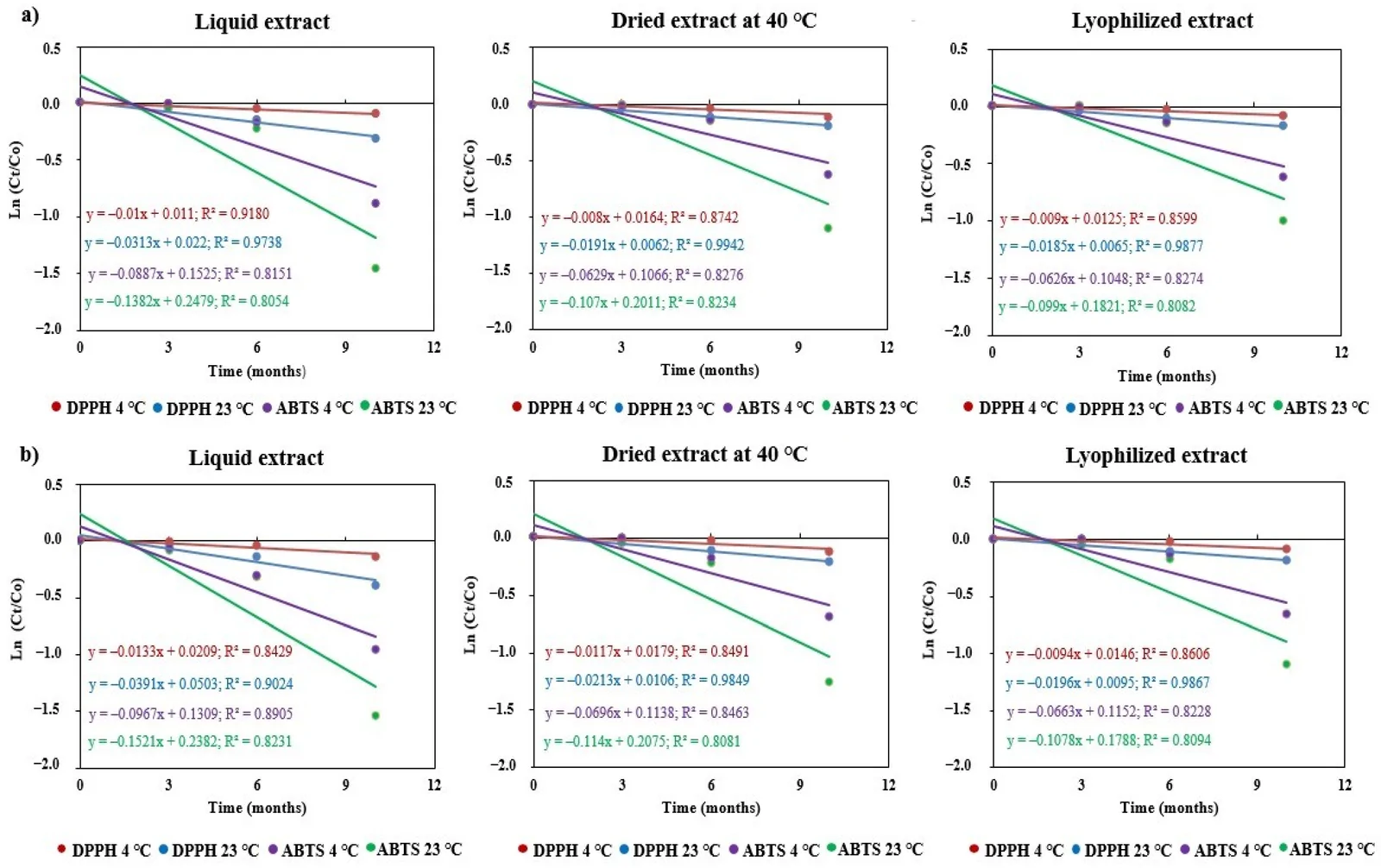
Degradation kinetics of AO activity of different forms of Cabernet Franc GSE (**a**) and Cabernet Sauvignon GSE (**b**), stored at 23 and 4 °C for 10 months.

The stability of polyphenols in dietary supplements and food additives for a long period of time is essential for their quality and health benefits. The kinetics of polyphenol degradation in GSE will provide further clarity on the extract stability. For this purpose, kinetic degradation models were studied during parallel storage of the extracts for 10 months at 23 °C and at 4 °C. Polyphenolic degradation was assessed by tracking the decline in antioxidant (AO) activity during sample storage. In order to determine which of the kinetic models is most suitable for describing the degradation process, determination coefficients R^2^ of the antioxidant capacities of the different extracts were first determined by plotting the graphs C_t_, lnC_t_, ln(C_t_/C_o_), 1/C_t_ and 1/C_t_^2^ against storage time at 23 and 4 °C, respectively, for zero, pseudo-first, first, second and third kinetic models [31,32]. It was found that the R^2^ for the first-order kinetic model had the highest values, ranging from 0.81 to 0.99, while for the other kinetic models, R^2^ was in the interval 0.65–0.82. Therefore, the first-order kinetic model is more suitable. Figure 4 presents the kinetic plots of AO activity degradation of all studied extracts during storage and corresponding equations.

The green line representing the ABTS antioxidant activity of the extracts stored at 23 °C shows greater experimental data scatter, resulting in the lowest R^2^ values (0.80–0.82). The lower R^2^ values may be attributed to the sharp decrease in ABTS antioxidant activity observed after 10 months of storage. The degradation profile of ABTS antioxidant activity in the extracts stored at 4 °C showed a better fit, with R^2^ values ranging from 0.82 to 0.89. These results may be attributed to the natural variability of the degradation process and the complex mechanism of action of the ABTS radical. Nevertheless, when R^2^ values exceed 0.80, the kinetic model can be considered to provide an acceptable fit to the experimental data [31,32]. In contrast, the DPPH antioxidant activity data for all extracts showed a very good fit to the first-order kinetic model, with R^2^ values ranging from 0.84 to 0.99.

The reaction rate constant (k), half-life (t_1/2_), time required for a 90% loss of AO activity (D), activation energy (E_a_), and the temperature coefficient (Q_10_), which describes the temperature dependence of the reaction rate constants, are presented in Table 5.

The obtained values for R^2^ in the range of 0.81 to 0.99 show that this model very well describes the kinetics of the loss of antioxidant activity of the extracts during their storage. Similar results have been found in the degradation kinetics of phenolics in grape stem extracts stored under different conditions [27]. The kinetic degradation constants (k) for the antioxidant activities determined by both DPPH and ABTS assays were lower in samples stored at 4 °C compared to those stored at 23 °C. Furthermore, the liquid samples exhibited higher k values than those of the corresponding dried extracts, irrespective of the drying method applied (oven drying at 40 °C or lyophilization). A substantial difference was observed between the kinetic parameters calculated from DPPH and ABTS antioxidant activities. The half-life time (t_1/2_) was defined as the time required for 50% degradation of the polyphenolic compounds in the extracts, corresponding to a 50% reduction in antioxidant activity. As expected, higher k values were associated with shorter t_1/2_ values, indicating faster degradation processes. Based on the DPPH assay results presented in Table 6, the liquid samples stored at 23 °C exhibited approximately threefold higher k values and, consequently, faster degradation rates compared to samples stored at 4 °C. In the dried samples, the corresponding increase was approximately twofold higher under storage at 23 and 4 °C. These findings indicate that degradation processes, evaluated on the basis of DPPH antioxidant activity, proceeded more rapidly and to a greater extent at 23 °C than at 4 °C. The kinetic parameters calculated from the ABTS assay further demonstrated higher k values compared to those obtained from the DPPH assay. Similarly, the degradation degree determined by the ABTS assay occurring at 23 °C was approximately 1.2 times faster than that observed at 4 °C, and the liquid concentrates again exhibited higher k values and lower t_1/2_ values than the dried samples. Comparison of the kinetic parameters between extracts derived from Cabernet Franc and Cabernet Sauvignon grape seeds revealed that the k values of Cabernet Franc extracts were consistently lower than those of Cabernet Sauvignon extracts. In contrast, the relationship observed for the half-life time (t_1/2_) was inverse. The activation energies (E_a_) of the degradation reactions were also calculated. As shown in Table 6, the activation energy values determined for the liquid samples were higher than those obtained for the dried extracts.

## 3. Discussion

Obtaining a grape seed extract with high antioxidant capacity and preserving its antioxidant potential for a longer period of time is a very important issue regarding its application as a dietary supplement and food additive. There are many factors that influence the stability of grape seed extracts. One of them is the residual moisture in grape seeds. A number of authors describe that residual moisture leads to degradation and changes in polyphenols in extracts [20,28]. The degree of moisture removal depends on the conditions applied for drying the seeds. From the experiments conducted, it is clear that the most suitable conditions are 40 °C, 24 h, as seen in Table 1. The residual moisture at 60 °C is the lowest, but the extract yield is the smallest compared to the other two methods, probably due to degradation of the more sensitive monomer molecules in the extract. This is also confirmed by some authors who describe that drying temperatures above 50–60 °C lead to degradation of the more unstable polyphenols [24]. The combined effect of moisture, light and temperature enhances oxidative and hydrolytic degradation processes [33,34,35].

As mentioned in the introduction, relatively few publications have investigated the stability of polyphenols in grape seed extracts over a long period of storage (data available for a maximum of 5–6 months) [27,33]. The polyphenol parameters studied (TPC, TF and PC) over a period of 10 months reflect the change in the stability of the extracts very well. The preservation of the polyphenol values shows that the extracts are stable for up to 3 months. Only at the 6th month is a reduction in the parameters noticeable. This reduction is smaller for dry samples stored at 4 °C, while the liquid samples stored at 23 °C exhibited substantially greater losses. A likely explanation for this observation is that the liquid concentrate is an aqueous solution, which leads to lower polyphenolic values due to the breakdown of the more unstable phenolic compounds. The negative effect of the aqueous medium on polyphenol stability has been discussed in a number of publications [19,36]. The values of the three polyphenol parameters are lowest in the samples stored for 10 months. Here too, the trend is maintained that the dry samples have a smaller loss than the liquid samples for all three parameters. At 4 °C, for 10 months, the losses of TPC, TF and PC in dried samples were 6.5%, 24%, and 10%, respectively, but in liquid samples, they were 15%, 30.6% and 18.06%. The losses of these parameters at 23 °C were higher: the TPC, TF and PC in dried samples were 12%, 45%, and 55%, respectively, but in liquid samples, they were 26.6%, 73.3% and 71.5%. Obviously, the longer time interval between 6 and 10 months is the reason for the greater drop in the studied indicators in this interval.

An interesting observation was the smaller decrease in TPC values compared to TF and PC values. This tendency was evident throughout the entire period of storage of the samples. These findings suggest that TF and PC, being more sensitive and less stable compounds, undergo faster degradation into lower-molecular-weight compounds during prolonged storage. These monomeric compounds are products of the oxidation and hydrolysis of the high-molecular-weight unstable flavonoids and procyanidins. The resulting monomers continue to contribute to the TPC values, which explains why this parameter did not exhibit a substantial reduction throughout the storage period, whereas TF and PC decreased significantly. Similar conclusions have been reported by some authors in previous studies [20,27].

For the stability of extracts, a very important factor is the grape variety from which the seeds are isolated, since the specific polyphenolic compounds and their different concentration define their resistance to heat, light, and oxidation. The concentration and structural characteristics of the phenolic compounds vary considerably among grape cultivars, and that is why the stability of each grape seed extract is distinct. External factors can affect the concentration and composition of flavan-3-ols in grapes, but genetic variation among grape varieties is regarded as one of the primary drivers of chemical differentiation in grapes and, consequently, in grape seeds [37]. In this study, the two investigated grape varieties were cultivated in the same geographical region (the area surrounding the town of Pomorie), under identical climatic conditions, and the grapes were harvested during the same vintage year (2025). Cabernet Franc and Cabernet Sauvignon are distinct wine grape cultivars, despite belonging to the same botanical family. It is known that Cabernet Sauvignon grapes are characterized by a high content of polymerized tannins, which genetically determines a higher stability of the extract [30,38]. However, the polyphenolic composition of the extracts depends greatly on the solvent used in their preparation. Since both extracts were obtained with a highly polar ethanol–water mixture, the extracted polyphenols are mainly phenolic acids and flavonoids (catechin, epicatechin, procyanidins B1–B4). Insoluble tannins cannot be extracted with this polar mixture.

Obreque-Slier et al. reported [30] that, at the end of seed maturation, most of the individual phenolic compounds in Cabernet Franc grape seeds were present at concentrations approximately twofold higher than those detected in Cabernet Sauvignon seeds. The only exceptions were procyanidins B1 and B2, whose concentrations were reported to be nearly identical in both grape varieties. The obtained HPLC results in the present study show a similar tendency. The differences observed in the polyphenolic parameters (TPC, TF and PC) of the extracts obtained from the seeds of the two grape varieties clearly confirm this distinction. The polymerized tannins were not extracted under the conditions used. Therefore, the observed variety-dependent differences in stability of the extract must be attributed to differences in the extracted soluble polyphenols rather than to insoluble tannins.

The influence of grape variety on the polyphenol stability of the extracts is best determined by identification and comparison of the individual composition of the two extracts during sample storage and by calculating the degree of degradation of phenolic compounds. To date, no studies have reported detailed investigations on the degradation of individual antioxidant compounds present in GSE over a storage period of 10 months. The available literature data are limited to storage periods of up to 5–6 months [18,20,27]. In the present study, HPLC analyses were performed on lyophilized and liquid extracts obtained from Cabernet Franc and Cabernet Sauvignon grape seeds at the initial stage (0 months) and after 10 months of storage at 23 °C and 4 °C. A comparative assessment of the degradation profiles of the identified compounds in extracts obtained from the two grape varieties was performed in order to determine whether grape cultivar influences extract stability. It is noteworthy that the initial concentrations of all identified individual compounds in Cabernet Franc extracts were higher than those detected in Cabernet Sauvignon extracts, with the exception of procyanidin C1.

It is noteworthy that the initial concentrations of all identified individual compounds in the Cabernet Franc extracts are higher than those found in the Cabernet Sauvignon extracts, with the exception of procyanidin C1. This trend is maintained at the end of the storage period of the extracts. The determined difference in the individual composition of the two extracts correlates very well with the obtained values of the studied polyphenol parameters (TPC, TF and PC) during extract storage. Therefore, HPLC analyses provide additional evidence that the higher content of extracted soluble polyphenols in the Cabernet Franc grape variety contributes to the greater stability of the extracts. Furthermore, these analyses revealed a slightly higher degree of degradation of individual phenolic components in Cabernet Sauvignon extracts at the end of 10 months compared to Cabernet Franc extracts. This difference is difficult to explain, as the degree of degradation depends not only on the total content of polyphenols, but also on their qualitative and quantitative composition, the content of galloylated flavan-3-ols and their resistance to oxidation.

A large difference in the concentration of gallic acid was observed between the initial extract and the extract stored for 10 months in both grape seed varieties. These two grape varieties are characterized by a very high content of gallic acid compared to other grape varieties [30,38]. It was found that this high concentration decreased rapidly, with losses reaching up to 55% after 6 months of storage. An additional loss of approximately 17 percentage points was observed between 6 and 10 months, resulting in a total loss of approximately 72%. Interestingly, in the liquid samples stored for 10 months (Figure 3), the higher storage temperature (23 °C) did not significantly affect the degradation of gallic acid, since its loss degree is the same as that of liquid samples stored at 4 °C. There was no significant difference in gallic acid degradation between the samples in liquid and lyophilized form. The comparison of the results obtained for samples stored at 23 °C and 4 °C at 10 months showed that neither the physical form of the extract nor the higher storage temperature significantly affected the degradation of the residual concentration of gallic acid. Some authors describe that gallic acid was reduced to a lesser extent compared to other polyphenols [27,39]; others indicate that upon the degradation of high-molecular-weight “galloylated” proanthocyanidins, gallic acid is released, and the gallic acid content in the stored extract increases [20,29]. According to our results, gallic acid underwent significant degradation within the first 6 months of storage in both grape seed extract varieties. The high initial content of gallic acid in these extracts may have contributed to its greater extent of degradation. After 6 months, the rate of degradation decreased. Gallic acid was the only compound for which no significant effect of extract physical form (liquid or dried form) or storage temperature (23 or 4 °C) on its loss over time was observed, suggesting that its degradation was relatively independent of these factors and indicating a certain degree of stability. For the other identified individual compounds, the results differ significantly. It has been found that the degradation degree of their liquid forms stored at 23 °C increases approximately 3–4 times compared to that of lyophilized samples stored at 4 °C.

In addition, Figure 3 clearly highlights the compounds exhibiting the highest degradation sensitivity, namely epicatechin, procyanidin C1, and procyanidin B3. The powerful antioxidants procyanidin B1, procyanidin B2, and catechin in lyophilized Cabernet Franc extract stored at 4 °C for 10 months had losses of only 0.59%, 2.34% and 4.02%, respectively. These small losses convincingly demonstrate the very good preservation of the antioxidant activity of the studied extracts stored at 4 °C and in dry form for 10 months. The data presented in Table 3 further demonstrate that epicatechin was approximately twofold more unstable than catechin during storage. The geometric and energetic parameters indicate that catechin is more stable than epicatechin [40]. The position of the hydroxyl group on ring C of the catechol moiety plays an important role in determining this relative stability. The comparison also clearly demonstrates the lower degradation losses observed in the lyophilized samples relative to the liquid extracts, as well as in samples stored at 4 °C compared to those stored at 23 °C. The obtained results for the dried extracts at 40 °C were very similar to those of the lyophilized samples. The TPC values of the Cabernet Franc sample oven-dried at 40 °C were only 2.5% lower than those of the lyophilized sample: the TF value was 0.5% lower, and the PC values were 0.74% lower at the end of storage (10 months, at 23 °C). The lowest degradation losses were consistently recorded in the two forms of dried samples stored under refrigerated conditions (4 °C, 10 months). The TPC losses do not exceed 8.5% for dried forms and 15% for liquid forms. These results indicate that these storage conditions are the most suitable for preserving extract stability.

An additional noteworthy observation was the comparatively lower degradation of procyanidin B1 relative to the other procyanidins. According to their degradation susceptibility, the procyanidins could be arranged in the following order: procyanidin C1 > procyanidin B3 > procyanidin B2 > procyanidin B1. Procyanidin C1, a type-B trimer, is generally considered to be less stable than procyanidin dimers, such as B1, B2, and B3 [41]. Procyanidins B1, B2, and B3 belong to the group of B-type dimers, and they differ in their structural conformations and monomeric arrangements [41,42]. Despite their structural similarities, significant differences in their stability characteristics have been reported. Procyanidin B1 is considered to possess a more compact and sterically protected structure compared to the B2 and B3 isomers [42,43]. These theoretical considerations support the findings obtained in the present study, which indicate that procyanidin B1 exhibits the highest stability among the investigated procyanidins.

In Table 6, a comparison of our results with results obtained from other authors is presented.

It is obvious that not many studies have investigated the stability of grape seed extracts for a longer period than 6 months. Data on grape pomace extracts predominate. There are also no data on the influence of grape variety on the stability of extracts. When comparing the results in Table 6, it is seen that, according to the studies of other authors, catechin degrades less than epicatechin. At 6 months, when storing the samples at 4 °C, the degradation of catechin is about 9.8% [21], and at 3 months, it is 7.98% [28], while in our studies, it is 4.06% at 10 months. For epicatechin, other authors present a 17.5% loss at 6 months [21] and 12.73% at 3 months [28]. According to our experiments, the percentage loss when storing the samples at 4 °C is 8.44% at 10 months. The degradation of procyanidins is in the same order as we have established: B3 > B2 > B1, but our percentages are lower. Other authors indicate that when storing the samples at 25 °C, the percentage losses of all studied components are much higher compared to those at 4 °C [22]. This is also confirmed in our studies. It is noteworthy that in some results, an increased content of gallic acid was noted, while in others, there was a percentage loss. An increased content of gallic acid was also found in our previous article [29], in which an extract of Syrah seeds was studied. These extracts probably contain a higher amount of galloylated flavan-3-ols and/or galloylated proanthocyanidins. During their degradation, free gallic acid is released, and an increased gallic acid content is reported [21,28]. It is obvious that the trends in the results presented in Table 6 are identical to those obtained by us. The variation in the percentages for individual parameters among different authors is due to the fact that there is a significant number of factors that influence the stability of polyphenols in the extracts—grape variety, drying conditions of the seeds or pomace, storage conditions of the samples, polyphenol composition of the extract and others.

**Table 6:** Comparison of the obtained results on the degradation of individual compounds in GSE with results from other authors.

Grape by-Product	Storage Time (Months)	Storage Conditions (°C)	Parameter Losses (%)	References
Negroamaro Grape Pomace, dried extract	6	25 °C	Gallic Acid—15.97% increase; (+)-catechin—15.69%; (−)-epicatechin—31.25%	[21]
		4 °C	Gallic Acid—1.30% increase; (+)-catechin—9.80%; (−)-epicatechin—17.50%	
Grape stem extracts of Mazuelo variety, lyophilised extract	6	25 °C	TPC—24.00%; AO (DPPH)—27.00%; Gallic Acid—24.00%; (+)-catechin degrade after 2 months.	[27]
Grape cane, dried extract	3	25 °C	Gallic Acid—52.00% increase; Procyanidin B1—17.22% increase; (+)-catechin—17.93%; Procyanidin B2—36.47%; (−)-epicatechin—25.95%	[28]
		5 °C	Gallic Acid—50.00% increase; Procyanidin B1—2.17% increase; (+)-catechin—7.98%; Procyanidin B2—11.76%; (−)-epicatechin—12.63%	
Syrah grape seed extract, lyophilized extract	10	23 °C	Gallic Acid—14.02% increase; Procyanidin B1– 1.35%; (+)-catechin—7.77%; Procyanidin B2—9.41%; (−)-epicatechin—22.33%; Procyanidin B3—11.48%; Procyanidin C1—26.02%	[29]
		4 °C	Gallic Acid—11.54% increase; Procyanidin B1– 0.68%; (+)-catechin—5.23%; Procyanidin B2—9.26%; (−)-epicatechin—16.21%; Procyanidin B3—9.84%; Procyanidin C1—26.02%	
Microencapsulateded grape pomace extract	2.5	25 °C, 33% relative humidities	Gallic Acid—2.41%; (+)-catechin—5.42%; (−)-epicatechin—2.13%; rutin—0.93%;	[36]
BRS Violet Grape Pomace, lyophilized extract	4	25 °C	TPC—21.38%	[44]
		4 °C	TPC—23.27%	
Grape marc from Albariño, liquid extract	2	20 °C	TPC—2.42% increase; AO (DPPH)—15.79%	[45]
		4 °C	TPC—3.29% increase; AO (DPPH)—13.68%	
Cabernet Franc grape seed extract, lyophilized extract	10	23 °C	Gallic Acid—76.24%; Procyanidin B1–0.91%; (+)-catechin—12.2%; Procyanidin B2—6.15%; (−)-epicatechin—23.54%; Procyanidin B3—22.26%; Procyanidin C1—32.78%	In this work
		4 °C	Gallic Acid—72.24%; Procyanidin B1– 0.61%; (+)-catechin—3.98%; Procyanidin B2—2.30%; (−)-epicatechin—8.44%; Procyanidin B3—17.42%; Procyanidin C1—22.85%	

The most important characteristic of dietary supplements is their antioxidant capacity. There is relatively good preservation of the antioxidant potential of the samples stored at 4 °C for 10 months (DPPH AO activity loss 11%). It was interesting that, after 10 months of storage, the reduction in antioxidant capacity determined by the ABTS assay was approximately threefold greater than that measured by the DPPH assay. This observation can be explained by the different reaction mechanisms underlying the two analytical methods [46,47]. The significantly larger decrease in antioxidant capacity detected after extended storage using the ABTS method, compared with the DPPH method, may be attributed to the degradation of high-molecular-weight polyphenolic compounds. These compounds are characterized by highly complex molecular structures and a strong ability to donate electrons, which allows them to interact very effectively with ABTS radicals [48]. However, during prolonged storage, they gradually degrade into lower-molecular-weight phenolic compounds, such as phenolic acids. Comparable degradation processes of complex polyphenols into phenolic acids during storage have been reported by many authors [20,49]. Phenolic acids possess hydroxyl groups, although fewer than proanthocyanidins and tannins, and are able to capture the DPPH radicals, thus preserving DPPH antioxidant activity to some extent. However, these acids show lower efficiency in electron transfer reactions with ABTS radicals. As a result, the antioxidant capacity, measured by the ABTS assay, decreases much more significantly during prolonged storage of the grape seed extracts.

The investigation of the kinetics of polyphenol degradation in GSE on the basis of ABTS and DPPH antioxidant activities provided further clarity on the stability of extracts and the selection of appropriate storage conditions for GSE. All calculated kinetic parameters clearly demonstrate that degradation processes were less pronounced in dried extracts stored under refrigerated conditions (4 °C). The liquid samples exhibited a higher kinetic degradation constant (k) than the corresponding dried extract, indicating that the liquid form of extracts causes a faster decrease in the contents of extract polyphenols. The results indicate that the bioactive components present in Cabernet Franc seed extracts degraded at a lower rate and more slowly than those in Cabernet Sauvignon extracts. The obtained kinetic parameters are identical to the results presented by other authors [27,50]. It is observed that the degradation rate constants associated with the ABTS assay were considerably higher than those determined by the DPPH method. This observation is consistent with the distinct reaction mechanisms underlying the two assays. During storage, high-molecular-weight polyphenolic compounds, which exhibit strong activity toward ABTS radicals, are progressively degraded into simpler phenolic structures [20,47]. These degradation products retain a certain capacity for scavenging DPPH radicals, while their effectiveness against ABTS radicals is markedly reduced.

The results presented in this study were obtained using a comprehensive set of analytical methods, kinetic models, and statistical analyses, providing a robust basis for evaluating the stability of the extracts and their potential application as food supplements. However, several limitations of the study should be acknowledged. The prolonged storage period also warrants monitoring of water activity and possible microbiological contamination, despite the appropriate handling and storage conditions of the samples. Furthermore, the present study was conducted using a single batch of seeds from each of the two grape varieties.

## 4. Materials and Methods

### 4.1. Materials

The materials used in this study were waste (seeds) from the vinification of red wine *Vitis vinifera* L. cv. Cabernet Franc and Cabernet Sauvignon. Fresh red pomaces of these grapes were provided in September 2025 by a Pomorie winery, Pomorie, Bulgaria. The two types of grapes are grown in the same geographic location, near the town of Pomorie, Bulgaria.

### 4.2. Chemicals

The solvent used for the preparation of GSE was ethanol 99.9% *v*/*v* (Valerus, Sofia, Bulgaria). The reagents for measuring polyphenolic parameters and antioxidant activity were methanol, gallic acid, 2N Folin–Ciocalteu reagent, sodium carbonate, sodium nitrate, aluminum trichloride, sodium hydroxide, hydrogen chloride, Vanillin, 2,2-diphenyl-1-picrylhydrazyl, 2,2′-azino-bis (3-ethylbenzothiazoline-6-sulfonic acid), and potassium persulfate, all purchased from Sigma-Aldrich Co., Steinheim am Albuch, Germany. The deionized water was purified by ELGA’s water purification systems (Lane End Business Park, HP14 3BY, UK). The reagents phosphoric acid, acetonitrile, (+)-catechin, (-)-epicatechin, procyanidin B1, procyanidin B2, procyanidin B3, and procyanidin C1 (HPLC-grade) were purchased from Sigma-Aldrich Co., Steinheim am Albuch, Germany, and used for HPLC analysis. All chemicals are analytical grade.

### 4.3. Preparation of Grape Seed Extract

**Figure 5:**
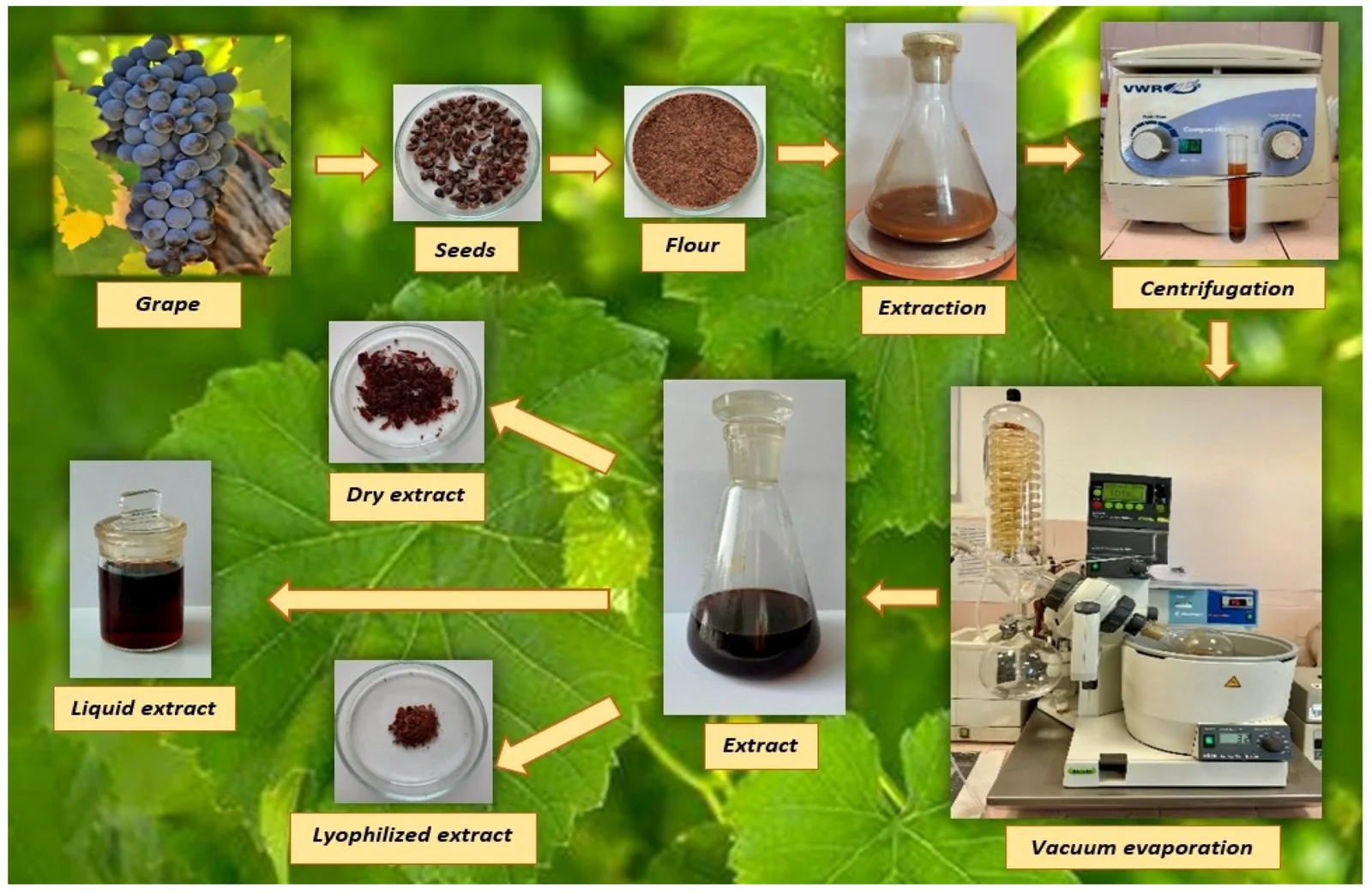
Stages of preparation of grape seed extract.

Seeds from the Cabernet Franc and Cabernet Sauvignon grape varieties were separated, cleaned of residual skins, washed, and dried either at room temperature (23 °C) for 10 days or in a laboratory dryer (Heraeus Acutherm, Hanau, Germany) at 40 °C for 24 h and 60 °C for 24 h. The stages of preparation of grape seed extract (GSE) are displayed in Figure 5.

The dried seeds were subsequently ground using a laboratory coffee grinder (MKM 7000, Bosch, Germany). The ground seeds (10 g) were mixed with 100 mL of 70% aqueous ethanol and subjected to extraction for 3 h on a magnetic stirrer (MMS-3000, Boeco, Hamburg, Germany). Then, the mixture was subjected to centrifugation at 6000 rpm for 10 min using a CompactStar CS 4 centrifuge (VWR, Equipnet, Boston, MA, USA). The supernatant was collected and concentrated using a rotary vacuum evaporator (Rotavapor R-215, Buchi, Flawil, Switzerland) at 45–50 °C under reduced pressure (100–175 hPa). The concentrated Cabernet Franc and Cabernet Sauvignon extracts were then divided into three equal portions. One portion was preserved as a liquid extract, a second portion was oven-dried at 40 °C for 24 h, and the remaining portion underwent lyophilization under controlled conditions. The lyophilization process was initiated at temperatures between −40 °C and −50 °C, followed by gradual adjustment to 0 °C under a pressure of 103 Torr. The drying process was finished at temperatures not exceeding 40 °C and under a pressure of 102 Torr for 4–8 h using a lyophilization system (VirTis, Los Angeles, CA, USA). Subsequently, all grape seed extracts (GSEs) were divided into two storage groups and stored either at 4 °C under refrigerated conditions or at 23 °C in the absence of light.

### 4.4. Total Phenolic Contents

The Folin–Ciocalteu method was employed to determine the total phenolic content, following the methodology of [51] with some modifications. The liquid extract (10 µL at a concentration of 200 µg/mL) was mixed with 990 µL of ethanol and homogenized using a vortex mixer. The dried extract 0.002 g was dissolved in a mixture of 300 µL of water and 700 µL of ethanol. The dilution factor of the samples was 0.01 (1:100). The samples were vortexed thoroughly and then centrifuged at 13,000 rpm for 15 min. Aliquots of 50 μL from the samples were mixed with 50 μL of 1N Folin–Ciocalteu reagent and 300 μL of sodium carbonate solution (75 g/L). After incubation in the absence of light for 30 min at room temperature, 2.6 mL of deionized water was added to the reaction mixture, and the absorbance was measured at 725 nm with a JENWAY 6900 UV–Vis spectrophotometer (Colmworth, UK). Measurements were performed in three independent replicates. Calibration curves were constructed using gallic acid standards (0.1–1 mg/mL) prepared in ethanol. The equation of the calibration line was: y = 0.0015x + 0.2041 and R^2^ = 0.9947. Results for total phenolic content were presented as milligrams of gallic acid equivalents per gram of dry weight extract (mg GAE/g dw).

### 4.5. Total Flavonoid Contents

Total flavonoids (TF) were determined based on the aluminum chloride colorimetric assay [52]. The liquid extracts (10 µL at a concentration of 200 µg/mL) were diluted with 990 µL of ethanol and thoroughly mixed using a vortex mixer. The dry and lyophilized extracts (0.002 g) were separately reconstituted in a mixture of 300 µL water and 700 µL ethanol. The resulting solutions were vortexed to ensure complete homogenization and subsequently centrifuged at 13,000 rpm for 15 min. An aliquot of 100 μL of each sample was mixed with 1 mL of distilled water and 75 μL of 5% sodium nitrite solution. After incubation for 5 min, 150 μL of 2% aluminum trichloride solution was added, and the mixture was incubated for an additional 6 min. Subsequently, 4 mL of distilled water and 500 μL of 1N sodium hydroxide were added, and the reaction mixture was subjected to incubation in the absence of light for 11 min. The absorbance of the resulting solution was measured at 510 nm using a JENWAY 6900 UV–Vis spectrophotometer (Colmworth, UK). All analyses were performed in triplicate. Calibration curves were constructed using quercetin standards in methanol within the concentration range of 0.1–1 mg/mL. The equation of the calibration line was: y = 0.7296x + 0.3504 and R^2^ = 0.9904. Total flavonoid content was expressed as milligrams of quercetin equivalents per gram of dry weight extract (mg QE/g dw).

### 4.6. Total Procyanidin Contents

Quantification of procyanidins was carried out according to the methodology described by Nakamura et al. [53], with some modifications. The liquid extracts, 100 µL at a concentration of 20 mg/mL, were diluted in 0.9 mL of ethanol and centrifuged at 13,000 rpm for 15 min; then, 2 mL of methanol was added. The dilution factor of the liquid samples was 1:30. Two milligrams of the dried and lyophilized extracts were dissolved in 1 mL of ethanol, centrifuged at 13,000 rpm for 15 min, and then 2 mL of methanol was added. The dilution factor of the two types of dried samples was 1:3. Reaction mixtures were prepared using different reagent combinations, mixtures were incubated at 30 °C for 20 min and then the absorptions were measured: A_o_ (1 mL methanol + 2.5 mL methanol and 2.5 mL 9 mol/L HCI); A_b_ (1 mL methanol + 2.5 mL 1% vanillin solution + 2.5 mL 9 mol/L HCl); A_c_ (1 mL GSE + 2.5 mL methanol + 2.5 mL 9 mol/L HCl); and A_s_ (1 mL GSE + 2.5 mL 1% vanillin + 2.5 mL 9 mol/L HCl). The absorbance of all mixtures was measured at 500 nm using a JENWAY 6900 UV–Vis spectrophotometer (Colmworth, UK). Calibration was achieved using catechin standards prepared in methanol over the concentration range of 20–800 µg/mL. The equation of the calibration line was: y = 0.0006x − 0.0189 and R^2^ = 0.9998. The procyanidin concentration was calculated and expressed as mg (+)-catechin equivalents per gram of dry weight extract (mg CE/g dw). All analyses were performed in triplicate. Absorption was calculated for each standard and sample solution as follows in Equation (1):

A = (A_s_ − A_b_) − (A_c_ − A_o_) (1)

### 4.7. High-Performance Liquid Chromatography (HPLC) Analysis of Extract Polyphenols

Reverse-phase HPLC analyses were conducted using a Dionex UltiMate 3000 UHPLC system equipped with a diode array detector (DAD) (Thermo Fisher Scientific, Waltham, MA, USA). Chromatographic separation was achieved on an Agilent Zorbax StableBond C18 column (250 × 4.6 mm, 5 µm) coupled with a corresponding Zorbax StableBond C18 guard column (12.5 × 4.6 mm, 5 µm), Agilent Technologies, Santa Clara, CA, USA. Gradient elution was applied using two mobile phases: solvent A, consisting of 0.1% phosphoric acid in water, and solvent B, consisting of acetonitrile (Table 7).

The injection volume was 10 μL, the flow rate was set at 0.7 mL/min, and the column temperature was maintained at 25 °C. Detection was carried out at 280 nm using a diode array detector. Prior to analysis, grape seed extracts were filtered through 0.45 μm membrane filters. Chromatographic peaks were identified by using standard solutions of polyphenolic compounds, including gallic acid, (+)-catechin, (−)-epicatechin, procyanidin B1, procyanidin B2, procyanidin B3, and procyanidin C1.

### 4.8. ABTS Antioxidant Capacity Assay

Antioxidant activity was assessed using the ABTS assay according to the method described by Sofi et al. [54]. The ABTS radical cation (ABTS•^+^) was produced by reacting equimolar amounts of 2.6 mM potassium persulfate and 7.4 mM ABTS solution, followed by incubation at room temperature under dark conditions for 16 h. Prior to analysis, the ABTS•^+^ stock solution was diluted with methanol to achieve an absorbance of 1.1 ± 0.02 at 734 nm. For the assay, 100 μL of sample solution was combined with 2 mL of the diluted ABTS•^+^ solution and incubated in the dark for 10 min. Subsequently, absorbance was recorded at 734 nm using a JENWAY 6900 UV–Vis spectrophotometer (Colmworth, UK). All determinations were performed in triplicate. Radical scavenging activity was calculated as a percentage using the following Equation (2)

% Inhibition of ABTS•^+^ activity = (A − B)/A × 100 (2)

A—the absorbance of the control; B—the absorbance of the sample.

### 4.9. DPPH Antioxidant Capacity Assay

Free radical scavenging activity was evaluated using the DPPH assay, according to the method described by Sofi et al. [54] with some modifications. An aliquot of 100 µL of each sample was mixed with 2 mL of freshly prepared 0.1 mM DPPH solution in methanol (2,2-diphenyl-1-picrylhydrazyl). The reaction mixture was incubated in the dark for 20 min. The absorbance was measured at 517 nm using a JENWAY 6900 UV–Vis spectrophotometer (Colmworth, UK). All samples were analyzed in triplicate. The percentage of radical scavenging activity (DPPH•^+^) was calculated using the following Equation (3).

% Inhibition of DPPH•^+^ activity = (A − B)/A × 100 (3)

A—the control absorbance; B—the sample absorbance

### 4.10. Polyphenol Degradation Model

Degradation kinetics were calculated based on the measured antioxidant capacity values of different grape seed extracts during the storage period [50]. The reaction rate constants (k) and half-life (t_1/2_) were determined assuming first-order kinetics using Equations (4) and (5).

ln (C_t_/C_o_) = − kt (4)

t_1/2_ = ln (2)/k (5)

C_o_ is the initial antioxidant capacity, and C_t_ is the antioxidant capacity at reaction time (t).

Using the selected reaction order, the activation energy (E_a_, kJ/mol) was estimated by an Arrhenius-type approach (Equation (6)), based on the slopes of linear plots of ln(k) versus 1/(T + 273.15) for each temperature. In this context, R denotes the universal gas constant (8.3144 J/mol·K), and T represents the storage air temperature (°C).

ln[k] = ln [A_o_] − [(E_a_/R) (1/(T + 273.15)] (6)

The Q_10_ quotient indicator was determined to describe the effect of temperature on the reaction rate constants, as shown in Equation (7).

Q_10_ = (k_2_/k_1_)**^(10/T_2_−T_1_)^** (7)

where k_1_ and k_2_ are the reaction rate constants, and T_1_ and T_2_ are storage temperatures.

D represents the time required for the compound or quality parameter to decrease by 90%, and it is calculated for first-order kinetics as shown in Equation (8).

D = 2.303/k(8)

where k (month^−1^) is the reaction rate constant.

### 4.11. Statistical Analysis

Statistical analysis was performed using a one-way analysis of variance (ANOVA) in SPSS software (SPSS Statistics version 19.0, IBM SPSS Statistics, Chicago, IL, USA) for all variables investigated in the study. Least squares mean (LSM) values were determined using Fisher’s least significant difference (LSD) test. All tests were conducted at a significance level of *p* < 0.05.

## 5. Conclusions

The stability of polyphenols and the antioxidant capacity of extracts obtained from valorized by-products, Cabernet Franc and Cabernet Sauvignon grape seeds, were evaluated over a long period of 10 months. The combined influence of grape variety, seed drying conditions, physical extract forms, and long-term storage conditions (10 months) on polyphenol stability was investigated. It was found that the most suitable seed drying conditions were 40 °C for 24 h. The effects of two different storage temperatures (23 and 4 °C) on the polyphenol stability of lyophilized, oven-dried (40 °C), and liquid forms of the extracts were evaluated over a 10-month period. The lyophilized Cabernet Franc GSE stored at 4 °C exhibited relatively high stability over the 10-month storage period, with losses of only 8.4% for TPC, 23.4% for TF, and 9.6% for PC. The oven-dried GSE stored under the same conditions showed similar stability, with slightly higher losses of approximately 1 percentage point for TPC and PC and 5 percentage points for TF. Similarly, the antioxidant activity of these extracts remained comparatively stable under refrigerated conditions, with decreases of only 7.63–8.17% in DPPH antioxidant activity. The Cabernet Franc GSEs showed a lower polyphenol loss than that observed for Cabernet Sauvignon GSEs in the investigated period. The degradation of the individual phenolic compounds during storage was also evaluated. The highest degradation losses after 10 months were observed for gallic acid, procyanidin C1 and epicatechin, followed by procyanidin B3. Procyanidin B1 exhibited the greatest stability among the polyphenolic compounds in lyophilized form stored at 4 °C (loss of only 0.6% for Cab. Franc GSE compared to 0.75% for Cab. Sauvignon GSE), followed by catechin (4.02% compared to 0.22%) and procyanidin B2 (2.35% compared to 5.04%). Overall, the obtained results clearly demonstrate that storage at 4 °C in dried extract form was the most appropriate condition for maintaining the stability of grape seed extracts. Furthermore, the high stability of antioxidant potential of the obtained extracts confirms their significant potential for application in the food, pharmaceutical, and cosmetic industries and in medicine.

## Figures and Tables

**Table 1:** The extract yield, grape seed moisture and TPC of extracts according to seed drying conditions.

Grape Variety	Seed Drying Temperature (°C)	Seed Drying Time (Days)	Moisture Content in Dried Seeds (% dw)	Yield (%)	TPC
	23	10	6.51 ± 0.06 ^a^	10.77 ± 0.93 ^a^	226.34 ± 0.31 ^b^
Cabernet Franc	40	1	6.33 ± 0.09 ^a^	11.89 ± 0.24 ^a^	230.71 ± 0.10 ^a^
	60	1	4.66 ± 0.08 ^a^	9.95 ± 0.21 ^a^	219.18 ± 0.09 ^c^
	23	10	6.91 ± 0.07 ^a^	11.81 ± 0.23 ^a^	210.47 ± 0.18 ^e^
Cabernet Sauvignon	40	1	6.65 ± 0.78 ^a^	12.23 ± 0.98 ^a^	215.66 ± 0.29 ^d^
	60	1	4.85 ± 0.91 ^a^	10.20 ± 0.31 ^a^	204.14 ± 0.06 ^f^

Standard deviation: mean ± SD (*n* = 3). Different superscripted lowercase letters within the same column indicate statistically significant differences among samples depending on the grape variety and the drying temperature of the grape seeds (*p* < 0.05), according to Fisher’s LSD test. % dw (% dry weight extracts).

**Table 2:** Polyphenolic values of different forms of grape seed extracts, stored at 4 °C and at 23 °C in a dark place according to the grape seed variety and storage conditions. Values in parentheses indicate the percentage decrease relative to the initial values of the polyphenols during storage for the corresponding months.

Storage Temperature	Storage Period	Parameters	Cabernet Franc			Cabernet Sauvignon		
			Liquid GSE	Dried GSE at 40 °C	Lyophilized GSE	Liquid GSE	Dried GSE at 40 °C	Lyophilized GSE
	0 month	TPC (mg GAE/g dw)	226.12 ± 0.47	237.07 ± 0.52	240.09 ± 0.21	210.60 ± 0.62	219.86 ± 0.65	220.92 ± 0.73
		TF (mg QE/g dw)	152.61 ± 0.37	161.72 ± 0.85	170.79 ± 0.63	140.72 ± 0.72	141.55 ± 0.67	148.92 ± 0.44
		PC (mg CE/g dw)	125.81 ± 0.59	130.87 ± 0.60	138.79 ± 0.63	112.80 ± 0.27	118.89 ± 0.49	120.11 ± 0.46
								
23 °C	3 month	TPC (mg GAE/g dw)	210.06 ± 0.71 ^a,B,^**	234.06 ± 0.27 ^a,A,^**	236.93 ± 0.27 ^a,A,^**	199.74 ± 0.98 ^a,B,^**	216.99 ± 0.78 ^a,A,^**	218.80 ± 0.30 ^a,A,^**
			(7.10%)	(1.27%)	(1.32%)	(5.16%)	(1.31%)	(0.96%)
		TF (mg QE/g dw)	144.68 ± 0.75 ^a,C,^**	156.04 ± 0.40 ^a,B,^**	167.06 ± 0.09 ^a,A,^**	132.57 ± 0.63 ^a,C,^**	137.96 ± 0.19 ^a,B,^**	147.08 ± 0.22 ^a,A,^**
			(5.19%)	(3.51%)	(2.19%)	(5.79%)	(2.54%)	(1.24%)
		PC (mg CE/g dw)	107.08 ± 0.16 ^a,B,^**	128.97 ± 0.13 ^a,A,^**	136.70 ± 0.28 ^a,A,^**	96.61 ± 0.53 ^a,B,^**	116.82 ± 0.71 ^a,A,^**	119.68 ± 0.62 ^a,A,^**
			(14.89%)	(1.45%)	(1.51%)	(14.35%)	(1.74%)	(0.36%)
							
	6 month	TPC (mg GAE/g dw)	205.17 ± 0.57 ^b,B,^**	230.07 ± 0.52 ^b,A,^**	233.26 ± 0.71 ^b,A,^**	190.94 ± 0.86 ^b,B,^**	214.01 ± 0.01 ^b,A,^**	216.14 ± 0.26 ^b,A,^**
			(9.27%)	(2.95%)	(2.83%)	(9.34%)	(2.66%)	(2.16%)
		TF (mg QE/g dw)	127.47 ± 0.21 ^b,C,^**	145.82 ± 0.28 ^b,B,^**	155.60 ± 0.42 ^b,A,^**	118.97 ± 0.49 ^b,C,^**	127.01 ± 0.05 ^b,B,^**	134.21 ± 0.30 ^b,A,^**
			(16.45%)	(9.83%)	(8.90%)	(15.46%)	(10.28%)	(9.88%)
		PC (mg CE/g dw)	94.08 ± 0.19 ^b,B,^**	115.28 ± 0.22 ^b,A,^**	120.71 ± 0.46 ^b,A,^**	85.92 ± 0.09 ^b,B,^**	104.90 ± 0.47 ^b,A,^**	107.62 ± 0.57 ^b,A,^**
			(25.22%)	(11.91%)	(13.03%)	(23.83%)	(11.77%)	(10.40%)
							
	10 month	TPC (mg GAE/g dw)	165.94 ± 0.54 ^c,B,^**	200.93 ± 0.40 ^c,A,^**	209.73 ± 0.69 ^c,A,^**	156.15 ± 0.42 ^c,B,^**	195.86 ± 0.81 ^c,A,^**	196.93 ± 0.40 ^c,A,^**
			(26.62%)	(15.24%)	(12.65%)	(25.85%)	10.91%	(10.86%)
		TF (mg QE/g dw)	40.57 ± 0.62 ^c,C,^**	87.92 ± 0.39 ^c,B,^**	92.64 ± 0.52 ^c,A,^**	39.93 ± 0.11 ^c,C,^**	79.82 ± 0.33 ^c,B,^**	80.72 ± 0.45 ^c,A,^**
			(73.29%)	(45.63%)	(45.76%)	(71.62%)	(43.61%)	(45.80%)
		PC (mg CE/g dw)	35.81 ± 0.12 ^c,B,^**	58.34 ± 0.15 ^c,A,^**	60.85 ± 0.25 ^c,A,^**	32.24 ± 0.49 ^c,B,^**	50.88 ± 0.28 ^c,A,^**	51.77 ± 0.39 ^c,A,^**
			(71.54%)	(55.42%)	(56.16%)	(71.42%)	(57.20%)	(56.90%)
								
4 °C	3 month	TPC (mg GAE/g dw)	221.19 ± 0.35 ^a,B,^*	234.17 ± 0.40 ^a,A,^*	237.42 ± 0.75 ^a,A,^*	204.43 ± 0.16 ^a,B,^*	217.99 ± 0.62 ^a,A,^*	219.59 ± 0.72 ^a,A,^*
			(2.18%)	(1.22%)	(1.11%)	(2.93%)	(0.85%)	(0.60%)
		TF (mg QE/g dw)	151.73 ± 0.24 ^a,C,^*	159.68 ± 0.51 ^a,B,^*	170.10 ± 0.35 ^a,A,^*	135.99 ± 0.04 ^a,C,^*	141.05 ± 0.23 ^a,B,^*	147.45 ± 0.33 ^a,B,^*
			(0.58%)	(1.26%)	(0.41%)	(3.36%)	(0.35%)	(0.99%)
		PC (mg CE/g dw)	120.93 ± 0.33 ^a,B,^*	129.76 ± 0.97 ^a,A,^*	137.57 ± 0.63 ^a,A,^*	108.92 ± 0.09 ^a,B,^*	117.81 ± 0.26 ^a,A,^*	119.62 ± 0.39 ^a,A,^*
			(3.88%)	(0.85%)	(0.88%)	(3.44%)	(0.91%)	(0.42%)
							
	6 month	TPC (mg GAE/g dw)	217.96 ± 0.36 ^b,B,^*	230.51 ± 0.68 ^b,A,^*	237.42 ± 0.75 ^b,A,^*	202.95 ± 1.00 ^b,B,^*	215.57 ± 0.16 ^b,A,^*	219.03 ± 0.07 ^b,A^
			(3.61%)	(2.77%)	(1.91%)	(3.63%)	(1.95%)	(0.86%)
		TF (mg QE/g dw)	140.89 ± 0.63 ^b,C,^*	153.50 ± 0.68 ^b,B,^*	162.57 ± 0.74 ^b,A^	128.50 ± 0.10 ^b,C,^*	131.10 ± 0.04 ^b,B,^*	135.89 ± 0.62 ^b,A,^*
			(7.67%)	(5.08%)	(4.81%)	(8.68%)	(7.38%)	(8.75%)
		PC (mg CE/g dw)	117.01 ± 0.11 ^b,B.^*	126.62 ± 0.40 ^b,A,^*	133.71 ± 0.26 ^b,A,^*	105.54 ± 0.45 ^b,B,^*	113.44 ± 0.30 ^b,A,^*	117.05 ± 0.42 ^b,A,^*
			(6.99%)	(3.25%)	(3.66%)	(6.44%)	(4.58%)	(2.55%)
							
	10 month	TPC (mg GAE/g dw)	192.09 ± 0.36 ^c,B,^*	216.90 ± 0.63 ^c,A,^*	219.88 ± 0.19 ^c,A,^*	180.16 ± 0.69 ^c,B,^*	208.09 ± 0.33 ^c,A,^*	210.08 ± 0.42 ^c,A,^*
			(15.05%)	(8.51%)	(8.42%)	(14.45%)	(5.35%)	(4.91%)
		TF (mg QE/g dw)	105.82 ± 0.12 ^c,C,^*	120.61 ± 0.70 ^c,B,^*	130.84 ± 0.06 ^c,A,^*	99.39 ± 0.54 ^c,C,^*	106.05 ± 0.09 ^c,B,^*	112.41 ± 0.54 ^c,A,^*
			(30.66%)	(25.42%)	(23.39%)	(29.37%)	(25.08%)	(24.52%)
		PC (mg CE/g dw)	103.09 ± 0.49 ^c,B,^*	118.23 ± 0.47 ^c,A,^*	125.51 ± 0.29 ^c,A,^*	93.51 ± 0.53 ^c,B,^*	106.00 ± 0.15 ^c,A,^*	107.48 ± 0.32 ^c,A,^*
			(18.06%)	(9.66%)	(9.57%)	(17.10%)	(10.84%)	(10.52%)

Standard deviation: mean ± SD (*n* = 3). Different superscripted lowercase letters within the same row indicate statistically significant differences (*p* < 0.05) among storage times (3, 6, and 10 months) for the same parameter and storage temperature. Values sharing the same lowercase letter are not significantly different. Different superscripted uppercase letters indicate statistically significant differences (*p* < 0.05) among the different GSE forms (liquid GSE, dried GSE at 40 °C, and lyophilized GSE) at the same storage time and temperature. Symbols * and ** indicate significant differences between storage temperatures (4 °C and 23 °C); both correspond to *p* < 0.05, with * denoting differences for samples stored at 4 °C and ** denoting differences for samples stored at 23 °C, according to Fisher’s LSD test. Percentages below each value indicate the loss of each parameter compared to the initial value. TPC (total phenolic content); TF (total flavonoids); PC (total procyanidins); mg/g dw (mg/g dry weight extract).

**Table 3:** Polyphenols in lyophilized and liquid Cabernet Franc and Cabernet Sauvignon grape seed extracts, analyzed by HPLC at 0 and 10 months, in mg/g expressed by dry weight extract (*n* = 3).

Grape Variety	Compounds	Initial Extract, 0 Month	Lyophilized Extract, Stored at 10 Months		Liquid Extract. Stored at 10 Months	
			23 °C	4 °C	23 °C	4 °C
Cabernet Franc	Gallic acid	7.66 ± 0.06	1.82 ± 0.33 ^a,A,^*	2.13 ± 0.34 ^a,A,^*	1.81 ± 0.02 ^a,A,^*	1.09 ± 0.34 ^a,A,^*
	Procyanidin B1	11.78 ± 0.17	11.67 ± 0.16 ^a,A,^*	11.71 ± 0.21 ^a,A,^*	10.63 ± 0.04 ^b,A,^*	10.55 ± 0.05 ^b,A,^*
	Procyanidin B3	0.60 ± 0.04	0.47 ± 0.01 ^a,A,^**	0.50 ± 0.02 ^a,A,^*	0.20 ± 0.01 ^b,A,^**	0.23 ± 0.02 ^b,A,^*
	(+)-Catechin	23.61 ± 0.37	20.72 ± 0.24 ^a,A,^*	22.66 ± 0.34 ^a,A,^*	9.93 ± 0.09 ^b,A,^*	10.56 ± 0.29 ^b,A,^*
	Procyanidin B2	6.83 ± 0.23	6.41 ± 0.09 ^a,A,^**	6.67 ± 0.04 ^a,A,^*	4.90 ± 0.05 ^b,A,^**	5.19 ± 0.04 ^b,A,^*
	(−)-Epicatechin	12.92 ± 0.13	9.88 ± 0.06 ^a,A,^**	11.83 ± 0.25 ^a,A,^*	3.47 ± 0.06 ^b,A,^**	5.11 ± 0.04 ^b,A,^*
	Procyanidin C1	1.01 ± 0.04	0.77 ± 0.01 ^a,A,^**	0.78 ± 0.01 ^a,A,^*	0.39 ± 0.02 ^b,A,^**	0.68 ± 0.01 ^b,A,^*
Cabernet Sauvignon	Gallic acid	4.06 ± 0.03	0.83 ± 0.04 ^a,B,^*	1.38 ± 0.08 ^a,B,^*	0.82 ± 0.02 ^a,B,^*	0.86 ± 0.02 ^a,B,^*
	Procyanidin B1	10.80 ± 0.02	10.01 ± 0.05 ^a,B,^*	10.56 ± 0.16 ^a,B,^*	9.47 ± 0.06 ^b,B,^*	9.60 ± 0.02 ^b,B,^*
	Procyanidin B3	0.54 ± 0.01	0.39 ± 0.03 ^a,A,^**	0.51 ± 0.07 ^a,A,^*	0.23 ± 0.00 ^b,A,^**	0.24 ± 0.00 ^b,A,^*
	(+)-Catechin	18.12 ± 0.04	13.80 ± 0.30 ^a,B,^*	18.08 ± 0.15 ^a,B,^*	10.78 ± 0.08 ^b,B,^*	11.28 ± 0.24 ^b,B,^*
	Procyanidin B2	6.35 ± 0.06	5.48 ± 0.04 ^a,B,^**	6.03 ± 0.04 ^a,B,^*	4.68 ± 0.05 ^b,B,^**	4.86 ± 0.06 ^b,B,^*
	(−)-Epicatechin	7.91 ± 0.04	4.37 ± 0.08 ^a,B,^**	5.99 ± 0.07 ^a,B,^*	2.44 ± 0.01 ^b,B,^**	3.17 ± 0.08 ^b,B,^*
	Procyanidin C1	1.23 ± 0.04	0.67 ± 0.08 ^a,A,^**	0.66 ± 0.01 ^a,A,^*	0.33 ± 0.03 ^b,A,^**	0.52 ± 0.03 ^b,A,^*

Standard deviation: mean ± SD (*n* = 3). Different superscripted lowercase letters within the same row indicate statistically significant differences (*p* < 0.05) among samples in different forms (liquid GSE and lyophilized GSE). Values sharing the same lowercase letter are not significantly different. Different superscripted uppercase letters indicate statistically significant differences (*p* < 0.05) among the different wine types (Cabernet Franc and Cabernet Sauvignon). Symbols * and ** indicate significant differences between storage temperatures (4 °C and 23 °C); both correspond to *p* < 0.05, with * denoting differences for samples stored at 4 °C and ** denoting differences for samples stored at 23 °C, according to Fisher’s LSD test.

**Table 4:** The antioxidant capacities (%) of different forms of grape seed extracts measured by ABTS and DPPH methods, stored at 4 °C and at 23 °C in a dark place. Values in parentheses indicate percentage decrease relative to the initial values of the antioxidant activity during storage for the corresponding months.

Storage	Storage Period	Parameters	Cabernet Franc			Cabernet Sauvignon		
			Liquid GSE	Dried GSE at 40 °C	Lyophilized GSE	Liquid GSE	Dried GSE at 40 °C	Lyophilized GSE
	0 month	DPPH (%)	81.98 ± 0.04	84.13 ± 0.13	84.74 ± 0.13	77.20 ± 0.06	79.08 ± 0.11	79.77 ± 0.38
		ABTS (%)	75.03 ± 0.26	76.41 ± 0.21	77.96 ± 0.23	74.91 ± 0.15	73.12 ± 0.17	74.95 ± 0.10
23 °C	3 month	DPPH (%)	78.35 ± 0.28 ^a,B,^**	80.74 ± 0.40 ^a,A,^**	81.84 ± 0.21 ^a,A,^**	74.30 ± 0.06 ^a,B,^**	75.82 ± 0.33 ^a,A,^**	76.93 ± 0.39 ^a,A,^**
			(4.43%)	(4.03%)	(3.42%)	(2.49%)	(4.12%)	(3.57%)
		ABTS (%)	71.58 ± 0.44 ^a,C,^**	72.99 ± 0.06 ^a,B,^**	77.58 ± 0.49 ^a,A,^**	68.23 ± 0.48 ^a,C,^**	69.81 ± 0.34 ^a,B,^**	70.79 ± 0.33 ^a,A,^**
			(4.64%)	(4.48%)	(0.49%)	(8.92%)	(4.53%)	(5.56%)
							
	6 month	DPPH (%)	70.20 ± 0.16 ^b,B,^**	75.75 ± 0.21 ^b,A,^**	75.72 ± 0.25 ^b,A,^**	66.84 ± 0.53 ^b,B,^**	71.10 ± 0.18 ^b,A,^**	71.65 ± 0.58 ^b,A,^**
			(14.37%)	(9.96%)	(10.64%)	(12.29%)	(10.10%)	(10.18%)
		ABTS (%)	58.28 ± 0.25 ^b,C,^**	61.72 ± 0.24 ^b,B,^**	64.98 ± 0.21 ^b,A,^**	54.85 ± 0.40 ^b,C,^**	60.32 ± 0.30 ^b,B,^**	62.94 ± 0.13 ^b,A,^**
			(22.33%)	(19.23%)	(16.66%)	(26.78%)	(17.51%)	(16.03%)
							
	10 month	DPPH (%)	60.64 ± 0.36 ^c,B,^**	69.86 ± 0.34 ^c,A,^**	70.92 ± 0.14 ^c,A,^**	51.87 ± 0.20 ^c,B,^**	64.07 ± 0.21 ^c,A,^**	65.89 ± 0.48 ^c,A,^**
			(26.04%)	(16.96%)	(16.31%)	(31.93%)	(18.99%)	(17.40%)
		ABTS (%)	16.87 ± 0.16 ^c,C,^**	25.83 ± 0.32 ^c,B,^**	28.90 ± 0.30 ^c,A,^**	16.05 ± 0.11 ^c,C,^**	20.93 ± 0.21 ^c,B,^**	24.90 ± 0.18 ^c,A,^**
			(77.52%)	(66.20%)	(62.93%)	(78.75%)	(71.38%)	(66.78%)
								
4 °C	3 month	DPPH (%)	81.68 ± 0.32 ^a,B,^*	83.94 ± 0.38 ^a,A,^*	84.63 ± 0.52 ^a,A,^*	75.85 ± 0.29 ^a,B,^*	78.80 ± 0.27 ^a,A,^*	79.58 ± 0.75 ^a,A,^*
			(0.37%)	(0.23%)	(0.13%)	(0.47%)	(0.35%)	(0.24%)
		ABTS (%)	74.20 ± 0.31 ^a,C,^*	76.00 ± 0.06 ^a,B,^*	77.19 ± 0.42 ^a,A,^*	70.93 ± 0.13 ^a,C,^*	72.72 ± 0.05 ^a,B,^*	74.82 ± 0.42 ^a,A,^*
			(1.11%)	(0.54%)	(0.99%)	(5.31%)	(0.55%)	(0.17%)
							
	6 month	DPPH (%)	78.78 ± 0.46 ^b,B^*	82.13 ± 0.42 ^b,A,^*	82.72 ± 0.55 ^b,A,^*	73.82 ± 0.29 ^b,B,^*	76.93 ± 0.25 ^b,A,^*	77.75 ± 0.64 ^b,A,^*
			(3.91%)	(2.38%)	(2.38%)	(3.13%)	(2.73%)	(2.53%)
		ABTS (%)	62.83 ± 0.48 ^b,C,^*	66.50 ± 0.06 ^b,B,^*	67.99 ± 0.34 ^b,A,^*	58.94 ± 0.13 ^b,C,^*	61.35 ± 0.42 ^b,B,^*	64.99 ± 0.18 ^b,A,^*
			(16.27%)	(12.97%)	(12.79%)	(21.32%)	(16.10%)	(13.29%)
							
	10 month	DPPH (%)	74.30 ± 0.06 ^c,B,^*	77.71 ± 0.42 ^c,A,^*	77.82 ± 0.26 ^c,A,^*	66.74 ± 0.40 ^c,B,^*	70.42 ± 0.19 ^c,A,^*	72.67 ± 0.61 ^c,A,^*
			(9.37%)	(7.63%)	(8.17%)	(12.41%)	(10.96%)	(8.90%)
		ABTS (%)	30.92 ± 0.18 ^c,C,^*	40.90 ± 0.27 ^c,B,^*	41.88 ± 0.23 ^c,A,^*	30.79 ± 0.33 ^c,C,^*	36.86 ± 0.22 ^c,B,^*	38.74 ± 0.54 ^c,A,^*
			(58.79%)	(46.47%)	(46.28%)	(58.90%)	(49.60%)	(48.31%)

Standard deviation: mean ± SD (*n* = 3). Different superscripted lowercase letters within the same row indicate statistically significant differences (*p* < 0.05) among storage times (3, 6, and 10 months) for the same AO activity. Values sharing the same lowercase letter are not significantly different. Different superscripted uppercase letters within the same row indicate statistically significant differences (*p* < 0.05) among the different GSE forms (liquid GSE, dried GSE at 40 °C, and lyophilized GSE). Symbols * and ** indicate significant differences between storage temperatures (4 °C and 23 °C); both correspond to *p* < 0.05, with * denoting differences for samples stored at 4 °C and ** denoting differences for samples stored at 23 °C, according to Fisher’s LSD test.

**Table 5:** Degradation kinetics of antioxidant capacity of the liquid, dried at 40 °C and lyophilized Cabernet Franc and Cabernet Sauvignon extracts over 10 months storage at 23 °C and 4 °C.

Grape Type	Type of Extract	23 °C				4 °C				(23–4 °C)	
		k (Month^−1^)	R^2^	D (Month)	t_½_ (Month)	k (Month^−1^)	R^2^	D (Month)	t_½_ (Month)	E_a_ (kJ/mol)	Q_10_
	**DPPH antioxidant activity**										
Cabernet Franc	Liquid	0.031 ± 0.005	0.97	74.29 ± 0.53	22.36 ± 0.25	0.010 ± 0.002	0.92	225.78 ± 2.03	67.95 ± 0.49	44.27 ± 0.41	1.80 ± 0.15
	Dried at 40 °C	0.019 ± 0.001	0.99	121.85 ± 0.96	36.67 ± 0.35	0.008 ±0.001	0.87	284.32 ± 2.36	75.57 ± 0.51	40.53 ± 0.39	1.57 ± 0.17
	Lyophilized	0.019 ± 0.002	0.98	124.49 ± 1.02	37.46 ± 0.38	0.009 ± 0.001	0.86	261.70 ± 2.25	78.76 ± 0.56	37.62 ± 0.35	1.48 ± 0.14
	**antioxidant activity**										
	Liquid	0.138 ± 0.017	0.81	16.71 ± 0.55	5.03	0.089 ± 0.007	0.82	25.96 ± 0.17	7.81 ± 0.08	31.20 ± 0.35	1.46 ± 0.15
	Dried at 40 °C	0.107 ± 0.015	0.82	21.44 ± 0.58	6.45	0.063 ± 0.004	0.83	36.61 ± 0.29	11.02 ± 0.09	24.32 ± 0.31	1.33 ± 0.11
	Lyophilized	0.100 ± 0.012	0.81	23.24 ± 0.61	6.99	0.063 ± 0.003	0.82	36.50 ± 0.32	11.03 ± 0.09	20.58 ± 0.28	1.27 ± 0.12
Cabernet Sauvignon	**DPPH antioxidant activity**										
	Liquid	0.039 ± 0.005	0.90	58.90 ± 0.38	17.73 ± 0.11	0.013 ± 0.003	0.84	173.16 ± 1.92	52.13 ± 0.56	42.19 ± 0.49	1.77 ± 0.16
	Dried at 40 °C	0.021 ± 0.002	0.98	108.12 ± 0.52	32.54 ± 0.25	0.012 ± 0.002	0.84	198.53 ± 1.98	59.75 ± 0.55	30.14 ± 0.41	1.38 ± 0.13
	Lyophilized	0.020 ± 0.001	0.99	117.50 ± 0.59	35.36 ± 0.29	0.010 ± 0.001	0.86	245.00 ± 2.35	73.73 ± 0.67	30.53 ± 0.45	1.48 ± 0.12
	**ABTS antioxidant activity**										
	Liquid	0.152 ± 0.023	0.82	15.14 ± 0.15	4.56 ± 0.05	0.097 ± 0.008	0.89	23.81 ± 0.35	7.17 ± 0.08	23.20 ± 0.21	1.37 ± 0.11
	Dried at 40 °C	0.114 ± 0.021	0.81	20.25 ± 0.19	6.10 ± 0.07	0.069 ± 0.007	0.85	33.09 ± 0.37	9.96 ± 0.09	21.41 ± 0.19	1.30 ± 0.09
	Lyophilized	0.108 ± 0.018	0.81	21.27 ± 0.18	6.40 ± 0.07	0.066 ± 0.006	0.82	34.89 ± 0.38	10.50 ± 0.09	18.47 ± 0.18	1.30 ± 0.10

Standard deviation: mean ± SD (*n* = 3). k—reaction rate constant, t_½_—half-life time, D—quality criterion to lose 90% of AO activity, E_a_—activation energy and Q_10_—quotient indicator.

**Table 7:** Gradient mode for RP-HPLC analyses of polyphenols in grape seed extracts.

Time (min)	Eluent A (%)	Eluent B (%)
0–7	90	10
7–40	80	20
40–65	75	25

## Data Availability

The original contributions presented in this study are included in the article. Further inquiries can be directed to the corresponding author.

## Abbreviations

The following abbreviations are used in this manuscript:

ABTS	2,2′-azino-bis(3-ethylbenzothiazoline-6-sulfonic acid)
AO	Antioxidant activity
B1	Procyanidin B1
B2	Procyanidin B2
B3	Procyanidin B3
C	(+)-Catechin
C1	Procyanidin C1
CE	Catechin equivalents
DPPH	2,2-diphenyl-1-picrylhydrazyl
dw	Dry weight
E_a_	Activation energy
EC	(−)-Epicatechin
GA	Gallic acid
GAE	Gallic acid equivalents
GSE	Grape seed extract
PC	Procyanidins
QE	Quercetin equivalents
RP- HPLC	Reversed-phase high-performance liquid chromatography
SD	Standard deviation
TF	Total flavonoids
TPC	Total phenolic content
